# Ratiometric Molecularly Imprinted Particle Probes for Reliable Fluorescence Signaling of Carboxylate-Containing Molecules

**DOI:** 10.1021/acsami.4c09990

**Published:** 2024-09-04

**Authors:** Yijuan Sun, Kornelia Gawlitza, Virginia Valderrey, Biswajit Bhattacharya, Knut Rurack

**Affiliations:** Bundesanstalt für Materialforschung und -prüfung (BAM), Richard-Willstätter-Str. 11, 12489 Berlin, Germany

**Keywords:** BODIPY, molecularly imprinted polymers, core−shell particles, antihistamines, dual emission

## Abstract

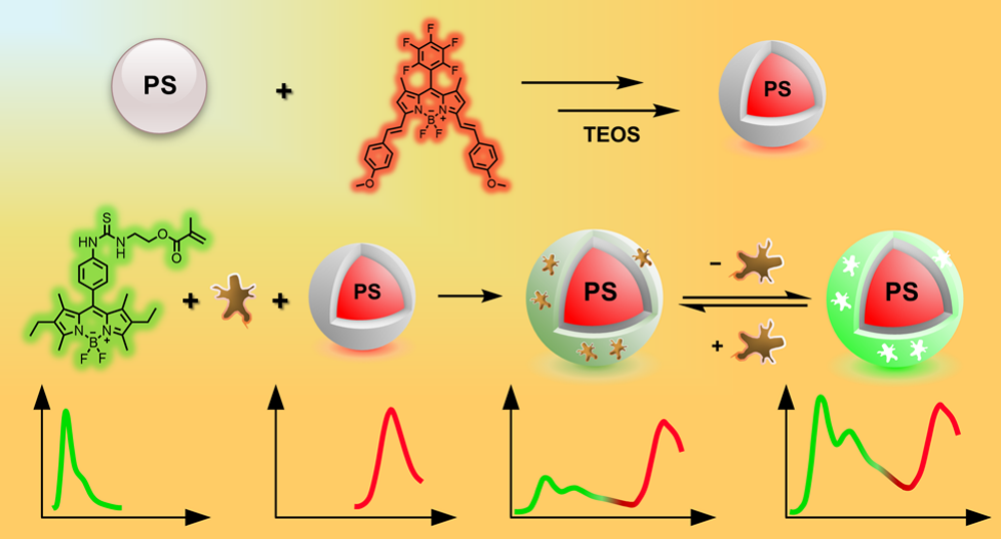

In addition to sensitivity, selectivity, and portability, chemical sensing systems must generate reliable signals and offer modular configurability to address various small molecule targets, particularly in environmental applications. We present a versatile, modular strategy utilizing ratiometric molecularly imprinted particle probes based on BODIPY indicators and dyes for recognition and internal referencing. Our approach employs polystyrene core particles doped with a red fluorescent BODIPY as an internal standard, providing built-in reference for environmental influences. A molecularly imprinted polymer (MIP) recognition shell, incorporating a green-fluorescent BODIPY indicator monomer with a thiourea binding site for carboxylate-containing analytes, is grafted from the core particles in the presence of the analyte as the template. The dual-fluorescent MIP probe detects fexofenadine as the model analyte with a change in green emission signal referenced against a stable red signal, achieving a detection limit of 0.13 μM and a broad dynamic range from 0.16 μM to 1.2 mM, with good discrimination against other antibiotics in acetonitrile. By selecting a versatile dye scaffold and recognition element, this approach can be extended to other carboxylate-containing analytes and/or wavelength combinations, potentially serving as a robust multiplexing platform.

## Introduction

Fluorescence analysis is a powerful technique for (bio)chemical sensing due to its high sensitivity, fast acquisition times, adaptable instrumentation, and versatility in using various luminescent materials, including quantum dots (QDs), metal–organic frameworks (MOF), metal nanoclusters, and organic dyes.^1^ Among organic dyes, boron-dipyrromethene (BODIPY) dyes stand out for their brightness, photostability, spectral tunability and synthetic versatility, allowing for the integration of a large number of functional substituents. BODIPYs are thus ideal candidates for fluorescent indicators or molecular probes in detecting a range of analytes such as metal ions, biologically active thiols or reactive oxygen, nitrogen and sulfur species.^2^

Despite the advancements in luminescent sensor materials, achieving selectivity for small molecule analytes remains challenging, particularly when biomolecular receptors like antibodies or aptamers are not available or cannot be used. To enhance target selectivity, integrating analyte-responsive fluorescent materials into polymer networks via molecular imprinting has emerged as a promising approach. Although various molecularly imprinted polymers (MIPs) incorporating polymerizable molecular probes or indicators have been developed,^3,4^ systems using responsive and polymerizable BODIPY dyes remain scarce.^5^

Molecular imprinting is a method for fabricating biomimetic materials with customized recognition sites.^6^ A target molecule, i.e., the analyte, is placed as a template in a mixture of functional monomers and/or cross-linkers that are complementary to the functional groups of the template in terms of noncovalent interactions. After polymerization and removal of the template, a molecularly imprinted polymer (MIP) is obtained whose cavities are complementary to the template not only in their functionality but also in their shape and size. MIPs are artificial receptors, making them particularly suitable for applications requiring chemical stability, solvent tolerance or the integration into robust sensing formats.^7−9^ Various MIPs incorporating fluorescent dyes, probes or indicators have recently been developed for detecting pesticides,^10−13^ drugs,^14−16^ proteins,^17−19^ and biomarkers.^20−22^ However, most existing approaches rely on modulating a single fluorescence signal for analyte detection, which can be problematic in onsite sensing scenarios due to potential interference from factors such as fluctuations in the excitation source, sensing matrix inhomogeneities, light scattering by the matrix, and microenvironmental changes. Dual-emission ratiometric measurements offer a solution to these challenges by comparing an analyte-sensitive signal with a stable reference signal at two distinct wavelength ranges, both excited at the same wavelength. This approach enables self-referencing, improves the signal-to-noise ratios, resulting in more reliable detection.^23,24^

Internally referenced dual-emission MIP-based sensing schemes involve combining two luminescent elements in a core–shell particle architecture. One element, shielded in the core, acts as a stable reference, while the other is responsible for recognition and signaling. For example, a system for detecting penicillin G (PenG) employs blue carbon dots (CDs) in a silica core for stable fluorescence, with yellow CDs in an imprinted polymer shell responding to PenG by quenching of the fluorescence.^25^ Similarly, ratiometric detection of 2,4-dichlorophenoxyacetic acid, okadaic acid or tumor-associated glycans utilizes red QDs or CDs as reference emitters and nitrobenzoxadiazole indicator monomers as signaling units.^26−28^ While most dual-emitting MIP systems combine QDs and/or CDs, or CDs and fluorescent dyes, fully dye-based systems are rare.^16,29^ This is surprising given the ease of doping polystyrene or silica core particles to adjust dye concentration precisely, as well as the straightforward use of indicator dyes in MIP layers, ensuring thin and homogeneous polymer shells for direct responses to analyte binding in an imprinted cavity.

This study introduces dual-emitting ratiometric MIP probes utilizing fluorescent BODIPY dyes for the reliable determination of a carboxylate-expressing drug. A red-emitting BODIPY dye is incorporated into polystyrene (PS) core particles as the internal reference, while a green-emitting BODIPY indicator monomer is covalently integrated into a thin MIP layer grown from an insulating thin silica shell, protecting the PS core. Fexofenadine (FEX), a second-generation antihistamine,^30^ was selected as the analyte/template due to its emergence as an environmental contaminant and as a representative carboxylate-containing drug.^31^ Antihistamines, crucial for treating allergic diseases affecting up to 40% of the global population, have recently become environmental contaminants due to increasing prescription rates and improper dosing.^32^ FEX, known for its efficacy, exhibits poor removal rates in wastewater treatment plants (<50%), resulting in residual concentrations that can exceed 0.1 μM in the environment.^33,34^ The development of robust, miniaturized, and portable detection methods directly deployable at the point-of-need is thus essential for effective management, circumventing delays associated with laboratory-based liquid chromatography–mass spectrometry (LC-MS),^35^ the prevailing technique for FEX determination.

## Experimental Section

### Reagents and Materials

All reagents were obtained from commercial suppliers and used without further purification unless otherwise noted. Spectroscopic toluene, chloroform (CHCl_3_), ethyl acetate (EtOAc), methanol (MeOH) and acetonitrile (MeCN) were purchased from Merck, anhydrous toluene and MeCN from Acros Organics. All other solvents including dichloromethane (CH_2_Cl_2_), ethanol (EtOH), and cyclohexane were supplied by Chemsolute. 2,4-Dimethyl-3-ethylpyrrole was purchased from J&K, triethylamine, 2,3-dichloro-5,6-dicyano-1,4-benzoquinone, pentafluorobenzaldehyde, 32% ammonia solution and tetraethyl orthosilicate (TEOS) were received from Merck, 2,4-dimethylpyrrole, 4-nitrobenzoyl chloride, 1,1′-thiocarbonyldiimidazole, *p*-anisaldehyde, boron trifluoride-diethyl etherate, 4-dimethylaminopyridine (DMAP), 2-isocyanatoethyl methacrylate, 4-cyano-4-(phenylcarbonothioylthio)pentanoic acid *N*-succinimidyl ester, styrene, 2,2′-azobis(2-methylpropionamidine)dihydrochloride (AIBA), 3-aminopropyltriethoxysilane (APTES), benzyl methacrylate (BMA), ethylene glycol dimethacrylate (EGDMA), tetrabutylammonium hydroxide solution in methanol (1 M), amoxicillin (AMOX) and ampicillin (AMPI) were obtained from Sigma–Aldrich, butylated hydroxytoluene (BHT) from Fluka, 2-aminoethyl methacrylate hydrochloride and aluminum oxide from Acros Organics, trifluoroacetic acid from Honeywell, 2,2′-azobis(2,4-dimethylvaleronitrile) (ABDV) from Wako Chemicals, and fexofenadine (FEX) from abcr. Milli-Q water was obtained from a Milli-Q ultrapure water purification system (Millipore Synthesis A10).

### Instruments

^1^H NMR, ^13^C NMR spectra were recorded on a Mercury 400 NMR spectrometer and referenced to the residual proton signals of the deuterated solvent. Ultrahigh-performance liquid chromatography electrospray ionization mass spectrometry (UPLC-ESI-MS) was acquired on an Acquity UPLC (Waters) with an LCT Premier XE time-of-flight mass detector (Waters). Absorption spectra were measured with a Specord 210 Plus spectrometer (Analytik Jena). Fluorescence spectra were recorded on a FluoroMax-4P spectrofluorometer (HORIBA Scientific). Fluorescence lifetimes were determined with a customized time-resolved laser setup consisting of a regenerative Ti:sapphire amplifier (Solstice Ace 100F10K HP, MKS Spectra Physics) and an optical parametric amplifier (TPR-Topas-F with TPR-NMW–UV1-F, MKS Spectra Physics) as excitation source as well as a spectrograph (Kymera 328i-A, Andor) and a streak camera setup (C1483–130, C13440–20CU, Hamamatsu) with electronics (C10647–10, C1097–05, Hamamatsu) as detection unit (further details on data fitting in Section I, Supporting Information). A time range of 20 ns was selected for recording the decays, corresponding to a time division of 55.3 ps per channel and yielding typical instrumental response functions of 250–300 ps (full width at half-maximum), and an uncertainty of measurement of ±30 ps. The laser beam was attenuated with a double prism attenuator from LTB and typical excitation energies were in the nanowatt-to-microwatt range (average laser power 0.5 mW). The fluorescence lifetime profiles were analyzed with the High-Performance Digital Temporal Analyzer (HPD-TA) software package including the TA-fit with the global deconvolution fitting module (Hamamatsu). Transmission electron microscopy (TEM) micrographs were imaged with a Talos F200S scanning/transmission electron microscope from ThermoFisher Scientific. Zeta potential measurements were carried out using a Zetasizer Nano-ZS from Malvern. Thermogravimetric analyses (TGA) were conducted on a STA200RV (Hitachi High-Tech Analytical Science) thermobalance.

Single crystal X-ray diffraction (SCXRD) data of crystals of compounds **M**_**1**_ and **M**_**2**_ were collected using a Bruker D8 Venture diffractometer (Bruker AXS) equipped with graphite-monochromated Mo Kα radiation (λ = 0.71073 Å). Data reduction was performed using the Bruker AXS SAINT and SADABS software packages.^36,37^ Direct method of SHELXT 2018 has been used to solve the structure of both crystals,^38^ followed by successive Fourier and difference Fourier syntheses. All hydrogen atoms bonded directly to carbon atoms were fixed at their ideal positions. Data collection, structure refinement parameters, and crystallographic data of both crystals are summarized in Table S1.

### Synthesis of *meso*-(4-Aminophenyl)-BODIPY 2 and Red BODIPY I

2,6-Diethyl-1,3,5,7-tetramethyl-8-(4-aminophenyl)-4,4-difluoro-4-bora-3a,4a-diaza-(*s*)-indacene **2** was prepared from *meso*-(4-nitrophenyl)-BODIPY **3** and 3,5-bis((*E*)-4-methoxystyryl)-8-pentafluorophenyl-1,7-dimethyl-4,4-difluoro-4-bora-3a,4a-diaza-(*s*)-indacene (red BODIPY **I**) for doping into polystyrene particles was obtained from 1,3,5,7-tetramethyl-8-pentafluorophenyl-4,4-difluoro-4-bora-3a,4a-diaza-(*s*)-indacene **II** as described in previous work.^39,40^ Details are given in Sections II, III, Supporting Information.

### Synthesis of *meso*-(4-Isothiocyanatophenyl)-BODIPY 1

1,1′-Thiocarbonyldiimidazole (250 mg, 1.4 mmol) was added to a solution of **2** (370 mg, 0.94 mmol) in CH_2_Cl_2_ (70 mL). The reaction mixture was stirred for 20 h at room temperature. The solvent was then evaporated, and the residue was chromatographed on silica gel using EtOAc/cyclohexane (1/5, v/v) as eluent to afford *meso*-(4-isothiocyanatophenyl)-BODIPY **1** as red crystals (356 mg, 87%). ^1^H NMR (400 MHz, CDCl_3_): δ (ppm) = 7.37 (d, *J* = 8.6 Hz, 2H), 7.30 (d, *J* = 8.6 Hz, 2H), 2.53 (s, 6H), 2.33 (q, *J* = 7.6 Hz, 4H), 1.30 (s, 6H), 1.00 (t, *J* = 7.6 Hz, 6H). ^13^C NMR (101 MHz, CDCl_3_): δ (ppm) = 154.47, 138.40, 138.10, 137.23, 135.08, 133.26, 132.25, 130.67, 130.04, 126.52, 17.20, 14.72, 12.67, 12.09, 12.04. HRMS-ESI: *m*/*z* calculated for C_24_H_26_BF_2_N_3_S [M]^+^: 437.1909, found: 437.1907.

### Synthesis of Indicator Monomer M_1_

2-Aminoethyl methacrylate hydrochloride (170 mg, 1 mmol) and triethylamine (0.19 mL, 1.37 mmol) were added to a solution of **1** (300 mg, 0.69 mmol) in CH_2_Cl_2_ (50 mL). The mixture was stirred at room temperature for ca. 85 h until total consumption of the starting material. The solvent was removed under reduced pressure, and the residue was purified by column chromatography on silica gel using EtOAc/CH_2_Cl_2_ (1/10, v/v) as eluent to give 2-(3-(4-(2,6-diethyl-1,3,5,7-tetramethyl-4,4-difluoro-4-bora-3a,4a-diaza-(*s*)-indacenyl)-phenyl)-thioureido)-ethyl methacrylate **M**_**1**_ as a red crystalline powder (369 mg, 95%). ^1^H NMR (400 MHz, CDCl_3_): δ (ppm) = 8.27 (s, 1H), 7.37 (d, *J* = 8.4 Hz, 2H), 7.34 (d, *J* = 8.5 Hz, 2H), 6.70 (s, 1H), 6.06 (s, 1H), 5.57 (s, 1H), 4.40 (t, *J* = 4.7 Hz, 2H), 4.04 (t, *J* = 4.7 Hz, 2H), 2.53 (s, 6H), 2.32 (q, *J* = 7.4 Hz, 4H), 1.89 (s, 3H), 1.31 (s, 6H), 0.99 (t, *J* = 7.4 Hz, 6H). ^13^C NMR (101 MHz, CDCl_3_): δ (ppm) = 180.77, 167.62, 154.35, 138.63, 138.07, 137.00, 135.88, 134.42, 133.20, 130.79, 130.41, 126.52, 124.70, 63.18, 45.55, 18.39, 18.36, 17.18, 14.70, 12.65, 12.02, 11.98. HRMS-ESI: *m*/*z* calculated for C_30_H_37_BF_2_N_4_NaO_2_S [M + Na]^+^: 589.2596, found: 589.2562.

### Synthesis of Indicator Monomer M_2_

*meso*-(4-Aminophenyl)-BODIPY **2** (280 mg, 0.71 mmol) and 4-dimethylaminopyridine (87 mg, 0.71 mmol) were dissolved in CH_2_Cl_2_ (20 mL) under argon, after which 2-isocyanatoethyl methacrylate (0.15 mL, 1.06 mmol) and butylated hydroxytoluene (10 mg, 0.05 mmol) were added. The reaction solution was stirred and refluxed at 50 °C for 48 h. The solvent was evaporated under vacuum, and the crude product was purified using column chromatography on silica gel using EtOAc/cyclohexane (1/1, v/v) as eluent to yield 2-(3-(4-(2,6-diethyl-1,3,5,7-tetramethyl-4,4-difluoro-4-bora-3a,4a-diaza-(s)-indacenyl)-phenyl)-ureido)-ethyl methacrylate **M**_**2**_ as a red powder (218 mg, 56%). ^1^H NMR (400 MHz, CDCl_3_): δ (ppm) = 7.50 (d, *J* = 8.5 Hz, 2H), 7.17 (d, *J* = 8.5 Hz, 2H), 6.98 (s, 1H), 6.13 (s, 1H), 5.59 (t, *J* = 1.6 Hz, 1H), 5.36 (t, *J* = 5.6 Hz, 1H), 4.32 (t, *J* = 5.4 Hz, 2H), 3.63 (d, *J* = 5.5 Hz, 2H), 2.52 (s, 6H), 2.31 (d, *J* = 7.6 Hz, 4H), 1.94 (s, 3H), 1.33 (s, 6H), 0.98 (t, *J* = 7.5 Hz, 6H). ^13^C NMR (101 MHz, CDCl_3_): δ (ppm) = 167.81, 155.30, 153.76, 140.12, 139.75, 138.59, 136.10, 132.95, 131.18, 130.28, 129.23, 126.36, 119.78, 64.10, 39.88, 18.43, 17.20, 14.73, 12.63, 12.03. HRMS-ESI: *m*/*z* calculated for C_30_H_37_BF_2_N_4_O_3_ [M]^+^: 550.2927, found: 550.2927.

### Preparation of Silica-Shelled Polystyrene Core Particles Doped with Red BODIPY I (rCS)

Polystyrene (PS) core particles were prepared by surfactant-free emulsion polymerization with a cationic initiator AIBA according to a published procedure with some modifications^41^ and then doped with red BODIPY **I** via a particle swelling/deswelling process based on a previously reported procedure, affording red BODIPY-doped PS particles (**rPS**).^42^ The **rPS** particles were subsequently coated with a silica shell to afford red core–shell particles (**rCS**),^43^ before amino groups were grafted to their surface via APTES condensation (**a@rCS**).^44^ Details on the preparations are given in Sections IV–VI, Supporting Information.

For the further functionalization with a RAFT agent, **a@rCS** particles (390 mg) were dispersed in anhydrous MeCN (12 mL), and 4-cyano-4-(phenylcarbonothioylthio)pentanoic acid *N*-succinimidyl ester (200 mg, 0.53 mmol) was subsequently added. The mixture was allowed to continue stirring for 24 h. After the reaction was completed, the particles were washed twice with MeCN, Milli-Q water and EtOH, respectively, with intervening centrifugation at 9140 × *g* for 12 min to remove the solvent. Subsequently, the desired **raft@rCS** particles were dried in a vacuum.

### Preparation of Green BODIPY Monomer-Containing MIP Particle Probe (gMIP@rCS)

For ratiometric MIP probe synthesis, BMA (4.0 μL, 22.4 μmol), EGDMA (21.7 μL, 113 μmol) and **raft@rCS** particles (10 mg) were added to a mixture of indicator monomer **M**_**1**_ (0.85 mg, 1.5 μmol) and the tetrabutylammonium salt of fexofenadine (FEX.TBA, 2.23 mg, 3 μmol; for preparation, see Section VII, Supporting Information) in MeCN (2 mL), followed by the addition of ABDV (0.62 mg, 2.5 μmol). The solution was flushed with argon at 0 °C for 5 min, to eliminate oxygen, and subsequently subjected to polymerization at 50 °C for 3 h. After the reaction was completed, the resultant MIP particles were collected by centrifugation and rinsed twice with MeCN (9140 × *g*, 10 min). To remove the template FEX.TBA, the particles were thoroughly dispersed in a solution of MeOH and acetic acid (90:1, 1.8 mL), fitted onto a rotator for further incubation at 40 rpm for 50 min and then recovered by centrifugation (6931 × *g*, 10 min). This procedure was repeated before the particles were washed three times with MeCN by centrifugation cycles (6931 × *g*, 10 min). Thereafter, the **gMIP@rCS** particles were dried at 20 °C in a vacuum.

### Fluorescence Analysis

To assess the recognition capability of the ratiometric **gMIP@rCS** probe imprinted with FEX.TBA, fluorescence titration analyses were conducted at room temperature in quartz cuvettes (10 × 10 mm). A dispersion of the probe particles was prepared in MeCN (0.05 mg mL^–1^), along with an analyte solution of 3.4 mM concentration. Subsequently, the analyte solution was incrementally added to the suspension, until no further change in fluorescence was observed. After each addition, the suspension was stirred for 2 min prior to fluorescence measurement to ensure thorough contact with the analyte and avoid signal fluctuation from particle sedimentation.

The ability for specific recognition was evaluated through the discrimination factor (DF), which was determined as the difference in response of the **gMIP@rCS** probe toward FEX.TBA and a potential competitor Z according to
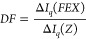
with
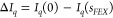
and
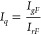
*I*_q_(0) is the green fluorescence intensity *I*_gF_ (at 538 nm) normalized by the red reference signal *I*_rF_ (at 690 nm) of the core in the absence of analyte and *I*_q_(s_FEX_) is the respective value at the saturation concentration of the target analyte (FEX). The DF is then the quotient of the two normalized difference signals for FEX and potential competitor (Z).

## Results and Discussion

### Design Considerations on Ratiometric BODIPY-Based MIP Particle Probe

According to our previous studies, fluorescent indicators—and to avoid confusion between “molecular probes” and “particle probes”, the term “indicator” is used for all “molecular probes” in this work—can be integrated into a MIP layer to facilitate monitoring of analytes of interest by a change in the fluorescence signal.^3^

To be suitable for incorporation into a MIP layer, the indicator needs to carry polymerizable unit(s), endure polymerization conditions, undergo analyte-induced spectroscopic changes, and produce a clear response even when immobilized under constrained conditions such as a cross-linked polymer matrix. In addition, a thin MIP layer on a core particle support not only enables faster diffusion of the template to the binding sites than in conventional monolithic MIP formats but also enhances the binding efficiency due to complete removal of the template and a more uniform distribution of the imprinted cavities.^3,11,20,22,45^

Given the versatility of BODIPY indicators and the applicability of a dual-emission MIP probe, we have designed two green BODIPY indicator monomers bearing (thio)urea receptors for complexing the target analyte FEX in its anionic form, along with a polymerizable unit for incorporation into a thin MIP shell.

As for the architecture of the particle probes, red BODIPY-doped polystyrene (**rPS**) particles have been selected as the core for the MIP sensor material, which exhibit a sharp red emission band centered at ca. 700 nm. If such fluorescent PS particles are covered with a thin silica shell, their dye-doped core is shielded from the environment during further functionalization as well as during analytical assays, providing a stable reference signal ideally suited for internally referenced, ratiometric measurements.

The third design criterion pertains to the fact that the choice of two brightly emissive entities allows to tailor the dual-fluorescent core–shell MIP probe in a way that it shows well-separated fluorescence bands of comparable intensity when excited with a single excitation wavelength. Such dual-emission sensing obtained using a single excitation wavelength simplifies the assay and effectively accounts for any changes in the analytical signal caused by factors other than molecular interactions such as, e.g., the drift of an excitation source. Well-separated emission bands further minimize emission crosstalk, contributing to reliability.

### Fluorescent Indicator Monomers

In the field of supramolecular chemistry, the development of indicators for the recognition of anionic species has attracted considerable attention due to their importance in biological processes, industrial applications and the environment.^46,47^ The perhaps most popular approach for the design of anion indicators involves the incorporation of (thio)urea motifs as receptors.

When fluorescent anion indicators are concerned, these motifs are at best integrated into the fluorophoric π system, constituting the signaling unit. The (thio)urea moiety features a planar Y-shaped six-atom arrangement [H–N–C(O/S)–N–H] able to provide two acidic hydrogens (N–H) for directional hydrogen bond-assisted binding of anionic species, especially those with a complementary Y-shaped motif such as carboxylates.

With respect to the optimal integration of the binding unit into the BODIPY fluorophore, we opted here for the attachment of the (thio)urea unit in *para*-position of a phenyl group introduced to the BODIPY scaffold in the *meso*-position instead of the recently reported direct attachment to the 3-position of one of the pyrrole rings.^5^

As discussed in detail below, one advantage of this strategy is that the typical narrow BODIPY fluorescence band is not significantly shifted or broadened by analyte binding, but only the fluorescence intensity is modulated, which is usually much more pronounced due to the special feature that *meso*-substituted BODIPYs allow a virtual decoupling of the receptor and fluorophore unit, entailing photoinduced electron transfer (PET)-like ON/OFF or OFF/ON responses.^48^ Another advantage is that the fluorescence quantum yields of the ON state of the indicator are high independent of solvent polarity, which is particularly advantageous when operating in highly polar and/or protic solvents in which charge transfer-type systems often show reduced emission.^48^

Therefore, we developed and synthesized the two fluorescent indicator monomers **M**_**1**_ and **M**_**2**_ to integrate the better performing one into our final system, dual-emitting, ratiometric MIP particle probes for the sensitive and selective fluorescence detection of a target analyte.

**Scheme 1:**
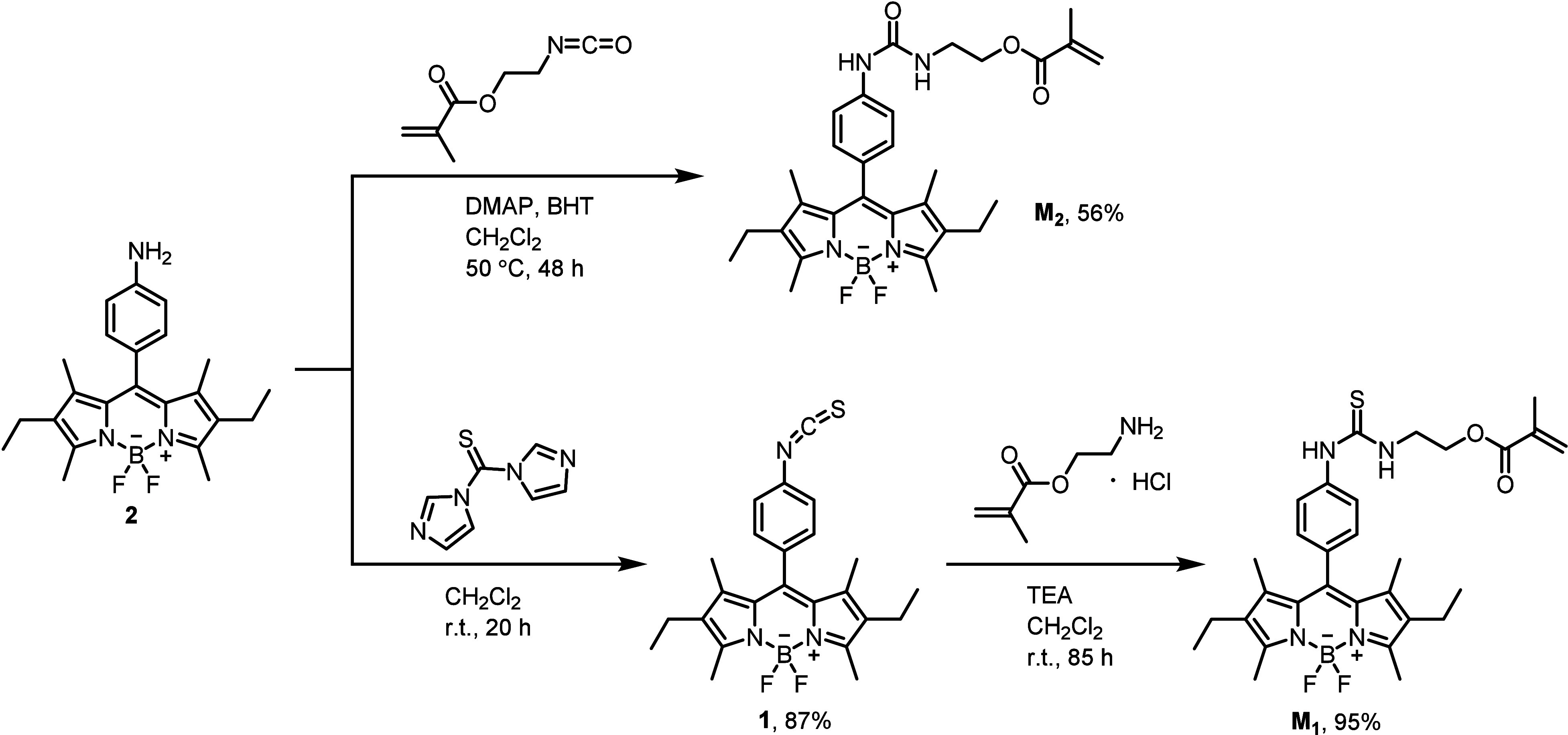
Synthetic Route for Fluorescent Indicator Monomers **M**_**1**_ and **M**_**2**_

The syntheses of **M**_**1**_ and **M**_**2**_ are depicted in Scheme 1. Precursor **2** was synthesized according to the literature from a nitro-substituted BODIPY in an optimized yield of 95%.^39^ The isothiocyanate intermediate **1** was obtained in 87% yield by treatment of precursor **2** with 1,1′-thiocarbonyldiimidazole (TCDI) in dichloromethane and was subsequently converted to indicator monomer **M**_**1**_ by reaction with 2-aminoethyl methacrylate hydrochloride in the presence of triethylamine (TEA).

The urea indicator **M**_**2**_ was prepared in 56% yield through a nucleophilic addition reaction of precursor **2** with 2-isocyanatoethyl methacrylate in the presence of 4-dimethylaminopyridine (DMAP) and butylated hydroxytoluene (BHT) at 50 °C in dichloromethane. Both indicator monomers were fully characterized by ^1^H NMR, ^13^C NMR, high resolution mass spectrometry (HR-MS) and X-ray single crystal analysis, see Supporting Information for more details.

**Figure 1:**
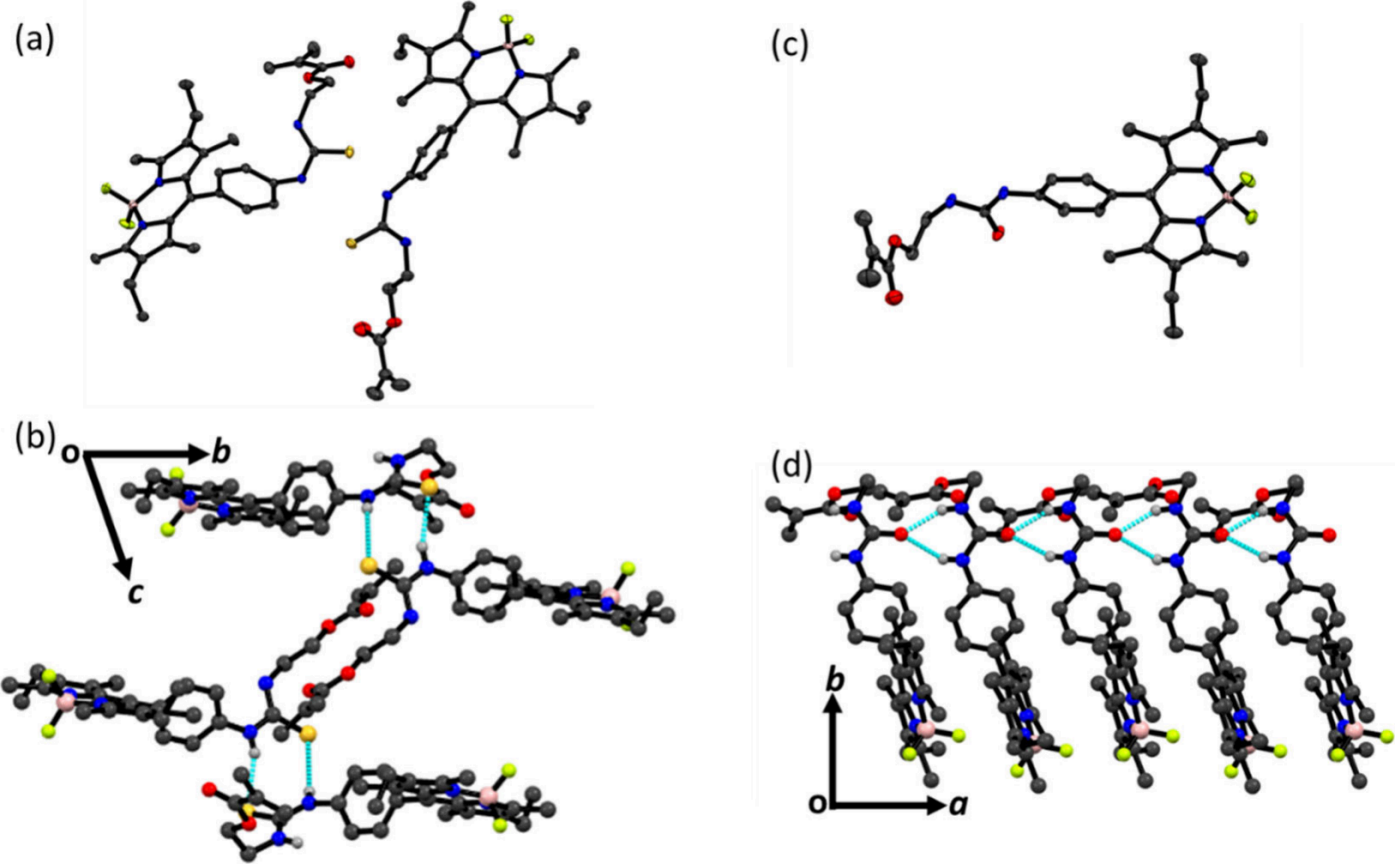
Crystal structure of **M**_**1**_ (a) and **M**_**2**_ (c) with the thermal ellipsoids plotted at the 40% probability, respectively. Molecular packing pattern in the crystal structure of **M**_**1**_ (b) and **M**_**2**_ (d), respectively. Cyan dotted lines show H-bonding interactions. Red for O, blue for N, bright green for F, magenta for B, yellow for S, and gray for C atoms. For clarity, hydrogen atoms and dichloromethane solvent molecule (in case of **M**_**2**_) are omitted.

The single crystals of indicator monomers **M**_**1**_ and **M**_**2**_ were obtained by slowly evaporating hexane into their dichloromethane solution. As shown in Figure 1 and Table S1, **M**_**1**_ crystallizes in the triclinic *P*1 space group with two molecules of **M**_**1**_, whereas **M**_**2**_ crystallizes in the orthorhombic *Pna*2_1_ space group with one molecule of **M**_**2**_ and one molecule of dichloromethane solvent. In both cases, the boron atom is coordinated by two nitrogen and two fluorine atoms in a tetrahedral geometry.

In crystal packing, **M**_**1**_ molecules are held together by two N–H···S motifs (*l*_H···A_, θ_N–H···A_: 2.430 Å, 160.10°; 2.733 Å, 153.18°), weak C–H···O and C–H···F interactions. In contrary, molecules of **M**_**2**_ are connected with each other by two N–H···O motifs (*l*_H···A_, θ_N–H···A_: 2.167 Å, 144.13°; 2.007 Å, 155.88°) and weak C–H···O and C–H···F interactions.

Furthermore, the photophysical behavior of the indicator monomers in different solvents was studied and the spectroscopic properties are compiled in Table 1. The absorption spectra of **M**_**1**_ and **M**_**2**_ show a narrow, intense S_1_ ← S_0_ transition in the visible region (at ca. 525 nm), a shoulder at the higher energy side at ca. 495 nm, assigned to the 0–1 vibrational band of the lowest-energy transition, and a weaker and broader S_2_ ← S_0_ transition at ca. 370 nm, which are typical absorption features of classical polymethine-type BODIPY dyes.

**Table 1:** Spectroscopic Properties of **M**_**1**_ and **M**_**2**_ in Selected Solvents at 298 K

Probe	Solvent	λ_abs_^a^ [nm]	λ_em_^b^ [nm]	ν̃_*abs-em*_^c^ [cm^–1^]	Φ_f_^d^	τ_f_^e^ [ns]	*k*_r_^f^[10^8^ s^–1^]	*k*_nr_^f^[10^8^ s^–1^]
**M**_**1**_	Toluene	529	540	385	0.72	4.11	1.7	0.7
	CHCl_3_	530	541	384	0.69	4.33	1.6	0.7
	EtOAc	522	533	395	0.63	4.05	1.6	0.9
	MeCN	523^g^	534	394	0.58	4.48	1.3	0.9
	MeOH	523	534	394	0.58	4.39	1.3	1.0
								
**M**_**2**_	Toluene	527	538	388	0.73	4.13	1.8	0.7
	CHCl_3_	527	538	388	0.76	4.45	1.7	0.5
	EtOAc	522	532	360	0.65	4.26	1.5	0.8
	MeCN	521	533	432	0.59	4.50	1.3	0.9
	MeOH	522	534	430	0.64	4.59	1.4	0.8

a Absorption maximum.

b Emission maximum.

c Stokes shift.

d For fluorescence quantum yield determination, see Section VIII, Supporting Information.

e Fluorescence lifetime.

f Radiative rate constants (*k*_r_) and nonradiative rate constants (*k*_nr_) were calculated using the following two equations: and .

g Molar absorption coefficient determined to 76695 ± 3842 M^–1^ cm^–1^ from *n* = 5 measurements.

**Figure 2:**
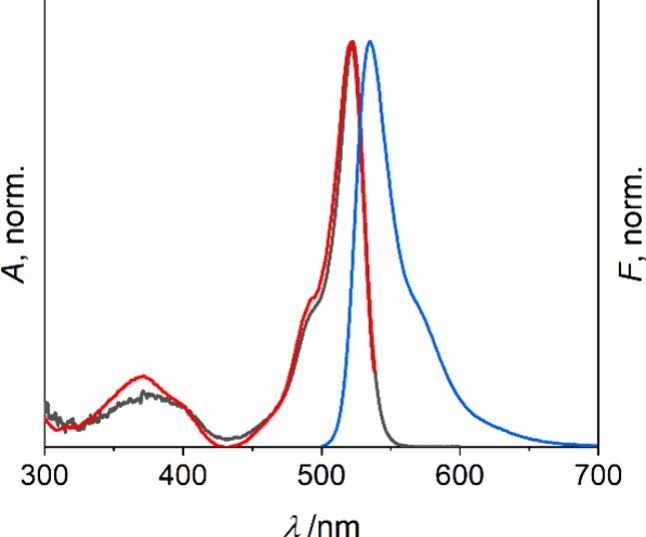
Absorption (black), fluorescence excitation (red, λ_em_: 538 nm, uncorrected) and fluorescence emission (blue, λ_exc_: 490 nm) spectra of **M**_**1**_ in MeCN.

The fluorescence excitation spectra match the absorption spectra which is exemplarily shown for **M**_**1**_ in Figure 2. The fluorescence emission spectra are mirror images of the S_1_ ← S_0_ absorption bands with Stokes shifts ranging from 360–432 cm^–1^.

There is no significant effect of solvent polarity on the absorption and emission maxima of **M**_**1**_ and **M**_**2**_, and both indicators are highly luminescent with fluorescence quantum yields ranging from 58–76%, which is desirable for ON/OFF-type fluorescence detection. Fluorescence lifetimes in the 4–5 ns range complement the data, yielding radiative rate constants *k*_r_ of ∼1.5 × 10^8^ s^–1^ and nonradiative rate constants *k*_nr_ of ∼0.9 × 10^8^ s^–1^ (Table 1), which are in good agreement with unquenched BODIPY dyes.^49^

The slight reduction of *k*_r_ depending on solvent polarity was also reported for other *meso*-(4-R-phenyl) BODIPYs and may be due to slight changes in the decoupling of both subunits by slight polarity-related changes in the mean torsion angle between BODIPY and *meso*-phenyl fragment.^50,51^

### Binding Behavior of Indicator Monomers at Dilute Concentration

The bright fluorescence of indicator monomers **M**_**1**_ and **M**_**2**_ in acetonitrile, in combination with hydrogen-bonding donor moieties on the molecule, render them suitable for indicating negatively charged carboxylates.

To examine the response of urea- or thiourea-equipped BODIPY monomers toward oxoanionic species, tetrabutylammonium acetate (AcO.TBA) was added to the corresponding acetonitrile solutions of the indicators, respectively.

**Figure 3:**
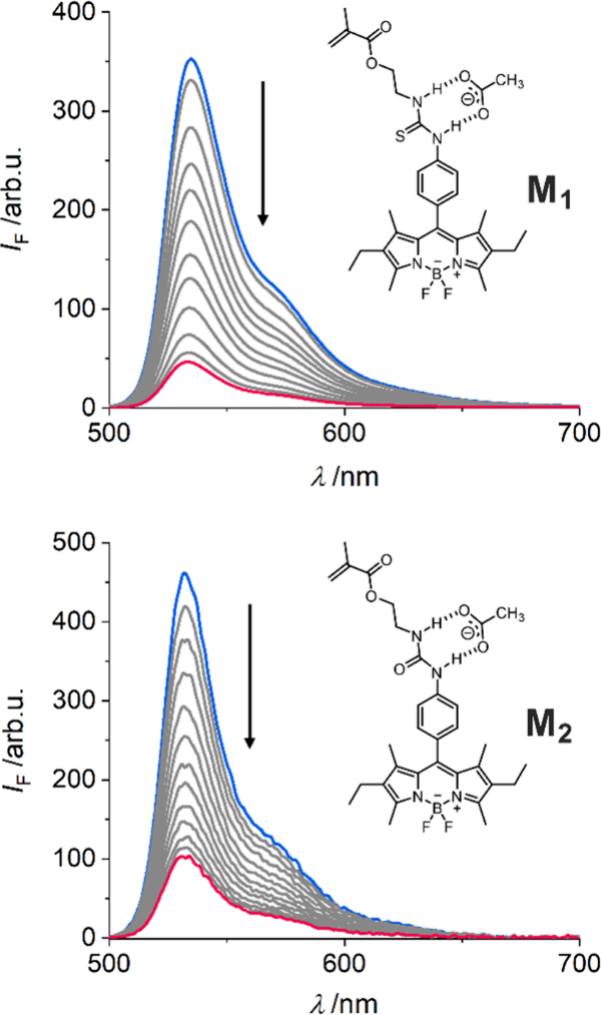
Fluorescence titration spectra of **M**_**1**_ (top) and **M**_**2**_ (bottom), *c*_**Mx**_ = 1 μM, with increasing concentration of AcO.TBA in MeCN (0–190 μM for **M**_**1**_, 0–150 μM for **M**_**2**_; start and end point spectra shown in blue and red), λ_exc_: 490 nm.

As shown in Figure 3, addition of AcO.TBA to a solution of **M**_**1**_ significantly reduced the fluorescence intensity, with a maximum quenching of up to 87% and a small but distinct hypsochromic shift of 2 nm at saturation. Meanwhile, a 2 nm blue shift as well as a slight increase of the absorption maximum can also be observed (Figure S1), suggesting hydrogen-bonding interactions between **M**_**1**_ and AcO^–^ (Figure 3), which is in agreement with *meso*-receptor-containing BODIPYs binding anions through H bonds.^52^

Similarly, incremental addition of AcO.TBA to a solution of probe **M**_**2**_ resulted in a 77% quenching of fluorescence, accompanied by a blue shift of 2 nm in the absorption maximum (Figure S1). Quantum chemical calculations carried out for **M**_**1**_ and **M**_**1**_⊂AcO^–^/TBA^+^ illustrate these experimental observations at the molecular level and suggest that a photoinduced electron transfer (PET) from the anion-bound *meso*-receptor phenyl unit is responsible for the quenching (Section XI including Figures S2, S3 and Tables S2, S3, Supporting Information).

The photophysical parameters of **M**_**1**_⊂AcO^–^/TBA^+^ reflect this interpretation with *k*_r_ = 1.1 × 10^8^ s^–1^ being only slightly reduced yet *k*_nr_ = 35.9 × 10^8^ s^–1^ being strongly increased as calculated from the complex’s Φ_f_ = 0.03 and τ_f_ = 0.27 ns, respectively. Both indicators are thus principally suitable for the detection of carboxylate-containing analytes through distinct changes in fluorescence intensity, with **M**_**1**_ being more sensitive than **M**_**2**_.

Furthermore, the binding constants of **M**_**1**_ and **M**_**2**_ with AcO.TBA were determined to *K*_**M1**⊂AcO.TBA_ = 5.23 (±0.01) × 10^4^ M^–1^ and *K*_**M2**⊂AcO.TBA_ = 2.76 (±0.01) × 10^4^ M^–1^ from fluorescence titrations, respectively (see Figure S4 for details). *K*_S_ of **M**_**1**_ is nearly twice that of **M**_**2**_, indicating that **M**_**1**_ exhibits not only greater sensitivity but also stronger binding affinity toward carboxylates. The stronger interaction between the analyte and the indicator monomer will provide a more stable analyte–indicator monomer complex prior to polymerization, contributing to polymers with higher imprinting efficiency.^53,54^

The aforementioned results make **M**_**1**_ a more promising indicator monomer for the fabrication of sensory MIPs with high affinity. However, because H-acidic thiourea-based indicators can be prone to deprotonation,^55^ which is not conducive to generating MIPs with good specificity and selectivity and cannot be easily distinguished from anion complexation for *meso*-receptor-substituted BODIPYs due to the small spectral shifts,^49,52^^1^H NMR titration studies of **M**_**1**_ were performed to obtain more insight into the interaction of **M**_**1**_ with carboxylates.

The ^1^H NMR spectrum of **M**_**1**_ in CD_3_CN showed representative thiourea protons (H^1^ close to phenyl, H^2^ close to ethyl) at δ 8.35 ppm and 6.91, respectively (Figure S5). Addition of 2 equiv. AcO.TBA to the solution of **M**_**1**_ produced downfield shifts of 4.58 ppm for H^1^ and 4.61 ppm for H^2^, indicating a deshielding of the two protons because of the formation of two directional hydrogen bonds with the highly electronegative acetate anion; a vanishing of (one of) the proton signals as would be indicative of deprotonation was not observed. **M**_**1**_ is therefore a promising indicator monomer for the construction of fluorescent MIP probes for the specific and selective detection of carboxylate-based analytes.

**Figure 4:**
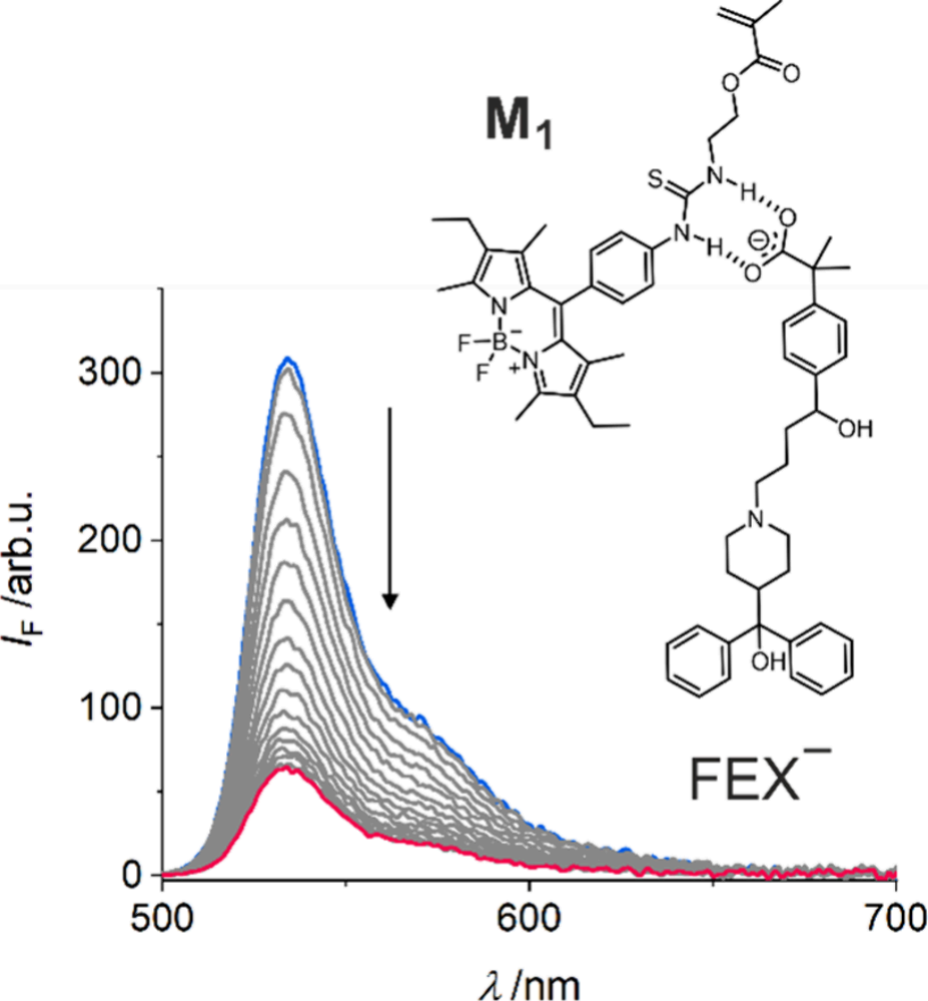
Fluorescence titration spectra of **M**_**1**_ (*c* = 0.7 μM) with increasing concentrations of FEX.TBA in MeCN (0–97 μM; start and end point spectra shown in blue and red), λ_exc_: 490 nm.

Upon establishing **M**_**1**_ as the indicator monomer of choice, we moved to our target analyte, fexofenadine (FEX). As expected, upon the addition of FEX.TBA, the fluorescence intensity of **M**_**1**_ in acetonitrile decreased significantly, resulting in a fluorescence quenching of up to 79% (Figure 4). Concomitantly, fluorescence quantum yield and lifetime were reduced to 0.04 and 0.28 ns, yielding a virtually unchanged *k*_r_ = 1.4 × 10^8^ s^–1^ but a strongly increased *k*_nr_ = 34.3 × 10^8^ s^–1^, characteristic for PET-type quenching.

The absorption changes were also found to be minor as in the case of AcO.TBA, Figure S6, Supporting Information. The corresponding binding strength was determined to *K*_**M1**⊂FEX.TBA_ = 5.70 (±0.02) × 10^4^ M^–1^ (Figure S6), which is slightly higher than that of AcO.TBA. This could be inferred as a result of slightly different electron densities of the two COO^–^ groups.

### Synthesis and Characterization of Ratiometric Core–Shell MIP Probes

To realize efficient and rapid fluorescence detection of analytes, few-nanometer thin molecularly imprinted polymer shells were grafted from submicrometric carrier particles, facilitating removal and rebinding of the template molecule. Thin MIP shells do not only offer rapid diffusion kinetics, but commonly also possess more uniformly distributed binding sites, favoring more efficient recognition of the template molecule.

Commonly used solid support materials for such systems include silica particles, Fe_2_O_3_ particles, carbon dots, and semiconductor quantum dots.^56^ Alternatively, and apart from the precursor material being cheap and widely commercially available, the advantages of polystyrene (PS) beads are that they can be produced monodispersed in large batches with sizes ranging from tens of nanometers to several micrometers, they can be easily doped with many different fluorophores in a concentration of choice for the implementation of a reference signal or code^42−44,57−60^ and they are more stable in suspension due to their low density.

Compared with silica, the loading of fluorophores into PS beads is more flexible and feasible. However, PS particles were rarely reported as solid supports for MIP shells, which can be mainly attributed to unfavorable surface characteristics for chemical modification for further polymer growth and poor resistance against organic solvents; this drawback can yet be solved by coating with a thin primary silica shell.^22,44^

Accordingly, monodisperse PS beads were prepared here as a support material using an optimized emulsion polymerization approach and doped with a red BODIPY dye (**I**) by a solvent swelling procedure, serving as an internal reference for a ratiometric measurement scheme. Subsequent coating of the doped PS beads (**rPS**) with a silica layer protects the core from organic solvents, affording red core–shell particles (**rCS**) that could be further chemically functionalized in a straightforward manner.

Reversible addition–fragmentation chain transfer (RAFT) polymerization was then employed to grow MIP shells with well-controlled thickness from the surface of **rCS**, the RAFT approach being well suited for high-performance MIPs.^61^ To graft a RAFT functionality, the **rCS** particles were first modified with 3-aminopropyltriethoxysilane (APTES) introducing active amino moieties, which were further subjected to an amidation reaction with hydroxysuccinimide-modified 4-cyano-4-(phenylcarbonothioylthio) pentanoate, anchoring the RAFT agent to the surface.

The changes in surface charge of the corresponding particles induced upon each step of modification were examined by zeta potential measurements in Milli-Q-water at pH 5 (Figure S7). The surface potential of **rPS** amounted to +47.1 ± 0.2 mV, which was attributed to the protonated amino groups provided by the AIBA initiator used during the synthesis. Such a sizable surface charge is crucial for minimizing particle aggregation and facilitating the coating of silica due to the electrostatic interaction between the amino groups and the silanol groups formed through TEOS hydrolysis.

A subsequent reduction in surface charge of up to 87 mV suggests the formation of a silica barrier. The modification of APTES brought its net charge back to +27.8 ± 0.9 mV (**a@rCS**), suggesting a change in the chemical groups on the surface of the particles from hydroxyl to amino. A renewed surface charge reduction to −13.3 ± 0.2 mV indicates an efficient conversion of amino into RAFT groups in **raft@rCS**.

Thermogravimetric analysis (TGA) of **rCS** particles illustrates that the thermal degradation of the PS core begins at 380 °C, reaching a mass loss of ca. 26% at 470 °C, after which only a small amount of inert residue remained (1–1.6%, Figure S7).

For the construction of a ratiometric sensory MIP platform, our aim was to grow a fluorescent polymer shell from **raft@rCS** particles with **M**_**1**_ as the fluorescent indicator monomer and the target analyte FEX.TBA as the template. To improve the recognition ability of imprinted cavities toward FEX.TBA and avoid aggregation between dye monomers, adequate structural comonomers and cross-linkers need to be employed.

Here, benzyl methacrylate (BMA) was chosen to assist cavity formation through π–π interaction with the aromatic moiety of FEX.TBA and ethylene glycol dimethyl methacrylate (EGDMA) was selected as cross-linking reagent due to its high affinity for polar analytes and structural flexibility.^62^ Moreover, once established and before embarking on MIP synthesis, it is important to check whether the response observed at dilute concentrations in neat solvent is also retained at polymerization concentrations in the respective mixture, guaranteeing that the desired species, the hydrogen bonded complex between indicator monomer and template, is present during the imprinting process.

Figure S8, Supporting Information, shows that this is indeed the case, i.e., that the slight hypsochromic shifts and the fluorescence quenching initially observed in dilute solutions upon addition of the analyte were still present at elevated concentrations in the prepolymerization mixture. Furthermore, to achieve a robust response from a MIP, the selection of a stoichiometry of **M**_**1**_:FEX.TBA that maximizes the spectroscopic changes in the mixture comprising MeCN, BMA, and EGDMA is necessary, ensuring the creation of high-affinity binding sites.

This practical approach is more suitable for MIP synthesis due to substantial differences in both concentration ranges employed for polymerization and the composition of the system, as compared to the host–guest model studies mentioned earlier. Additionally, for MIPs designed for an optical response, it is also advantageous to engage all fluorescent indicator monomers in complexation in a well-defined manner at the outset of polymerization. To achieve these goals, a stoichiometric ratio of 1:2 between **M**_**1**_ and FEX.TBA promised to yield a more favorable outcome than 1:1 (Figure S8).

**Scheme 2:**
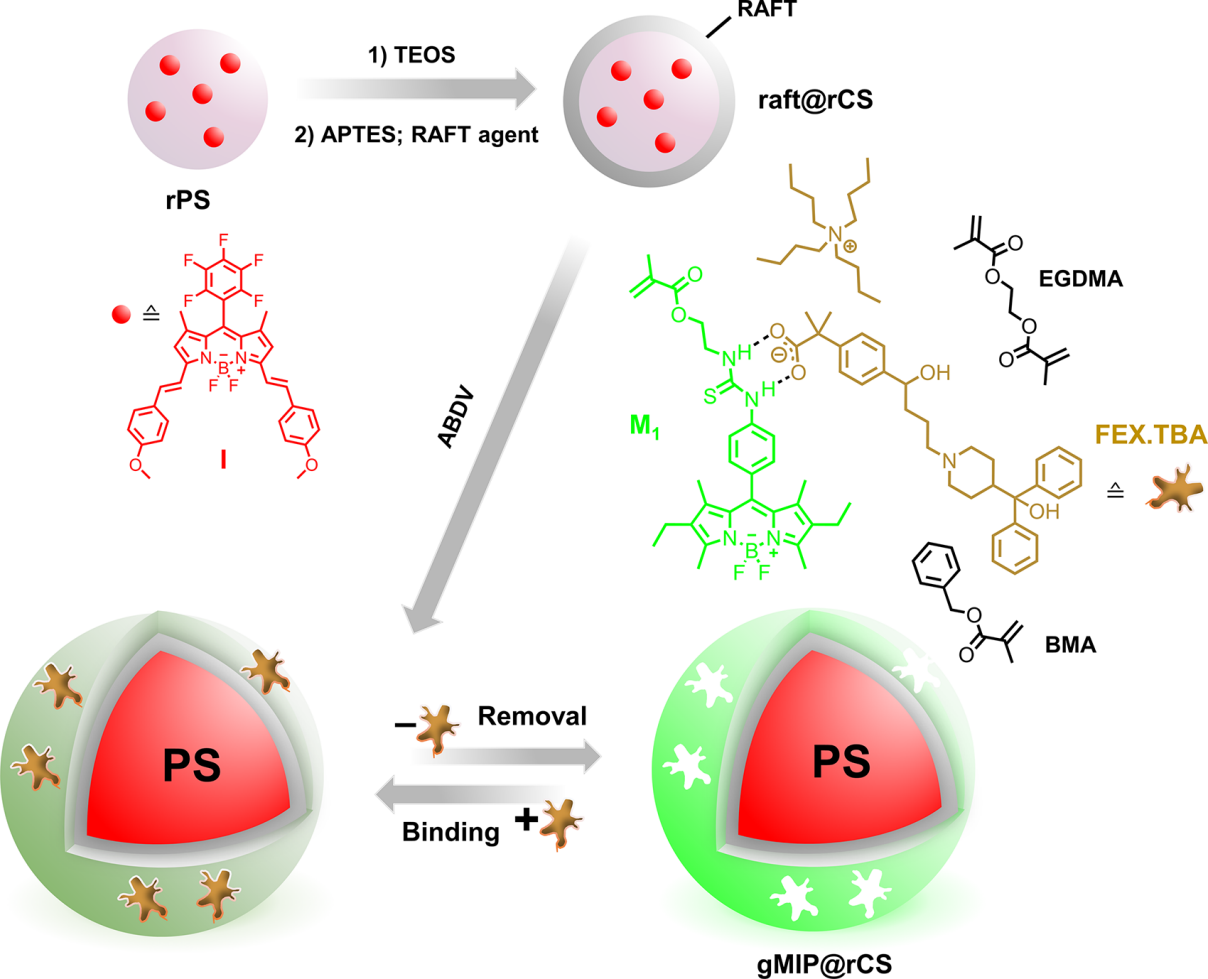
Schematic of Ratiometric Sensory MIP Probe Synthesis

Polymerization was then accomplished under the initiation of 2,2′-azobis(2,4-dimethylvaleronitrile) (ABDV), and the resulting particles were incubated in an acidic solution to release the template, yielding dual-emitting core–shell MIPs (**gMIP@rCS**, Scheme 2). Further details on specific aspects of MIP preparation are given in Sections XVIII and XIX, Supporting Information, including Table S4 and Figures S9, S10.

**Figure 5:**
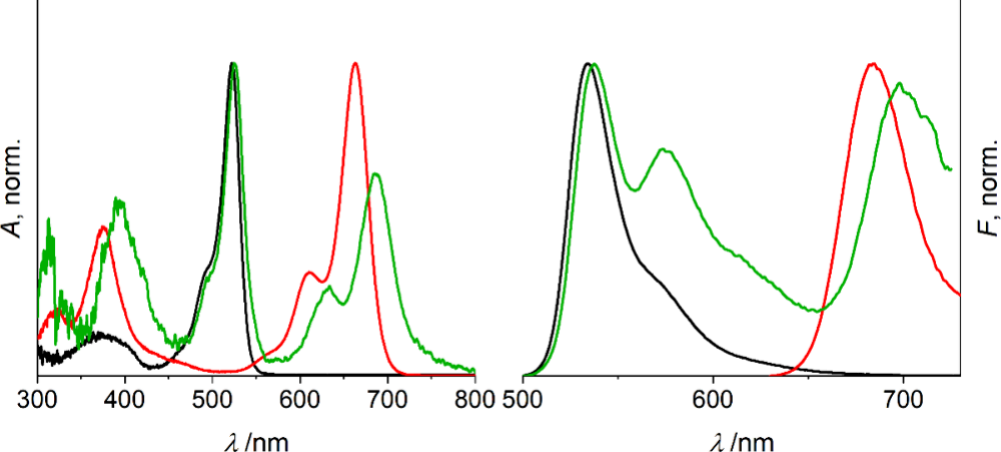
Absorption (left) and fluorescence emission (right) spectra of **M**_**1**_ (black), **I** (red) and **gMIP@rCS** (green) in MeCN. λ_exc_: 375 nm.

The successful integration of green indicator monomer **M**_**1**_ and red reference dye **I** was verified by absorption and emission spectra. As shown in Figure 5, the absorption maxima of **M**_**1**_ and **I** at diluted concentrations are located at 523 and 663 nm, respectively. Moreover, both BODIPY dyes show moderately intense and optimally overlapping absorption bands at 375 nm, suggesting that light sources within this range can readily excite both fluorescence colors of **gMIP@rCS**.

Because the molar absorption coefficient of **I** is favorably high (e.g., ε_663 nm_ = 120060 ± 2920 M^–1^ cm^–1^ in MeCN), it is still sizable at 375 nm (47%) and, together with a high Φ_f_ (e.g., 0.64 in MeCN), accounts for the commonly lower sensitivity of a detection system at >650 nm. The fluorescence emission maxima of **M**_**1**_ and **I** in diluted state can be observed at 534 and 684 nm, respectively, upon excitation at 375 nm.

In contrast, the resultant **gMIP@rCS** probe exhibits two main emission bands at 538 and 698 nm, with a sideband at 575 nm. As is detailed in Section XX, including Figures S11–S14, Supporting Information, a careful analysis of the spectroscopic behavior of **M**_**1**_ at different concentrations on the basis of the putative amount of **M**_**1**_ incorporated into the MIP shell revealed that a certain amount of **M**_**1**_ species in the polymer seems to be able to interact in the excited state, leading to a red-shifted, unstructured emission band with a maximum at ca. 590 nm reminiscent of excimers or exciplexes.

Because no hints were found for BODIPY dimers in the ground state, the spectral emission pattern of **gMIP@rCS** is reproducible, the 590 nm-band is only related to **M**_**1**_ and shows the same behavior upon anion binding as the characteristic monomer band at 534 nm, see below, further experiments to get a deeper understanding of the involved process were not attempted within the framework of this study. Compared to dilute solution, dye **I** encapsulated in the **rCS** core shows a red shift of 14 nm in emission and ca. 20 nm in absorption, which is due to dispersive interactions because of the high refractive index of the surrounding matrix (Figure S15). The reproducibility of the **gMIP@rCS** synthesis was demonstrated via three individual batches, being illustrated by virtually identical fluorescence emission spectra (Figure S16).

**Figure 6:**
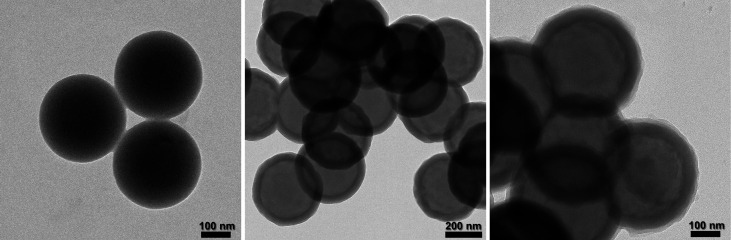
TEM images of **rPS** (left), **rCS** (center) and **gMIP@rCS** (right).

Transmission electron microscopy (TEM) analyses of **rPS**, **rCS** and **gMIP@rCS** particles were performed to examine the morphologies and structures of the particles. As shown in Figure 6, the TEM image of **rPS** showed monodisperse and spherical structures with an average diameter of 299 ± 6 nm. After coating with silica, a homogeneous shell was formed around the PS beads with a mean thickness of 46 ± 7 nm.

Furthermore, the successful growth of a MIP layer was confirmed by the presence of uniform polymer shells of 17 ± 4 nm, such that thin shells facilitate rapid and efficient rebinding of the template.

### Optosensing with gMIP@rCS

Upon the successful integration of **M**_**1**_ we further investigated the optosensing performance of the ratiometric **gMIP@rCS** probe for FEX.

**Figure 7:**
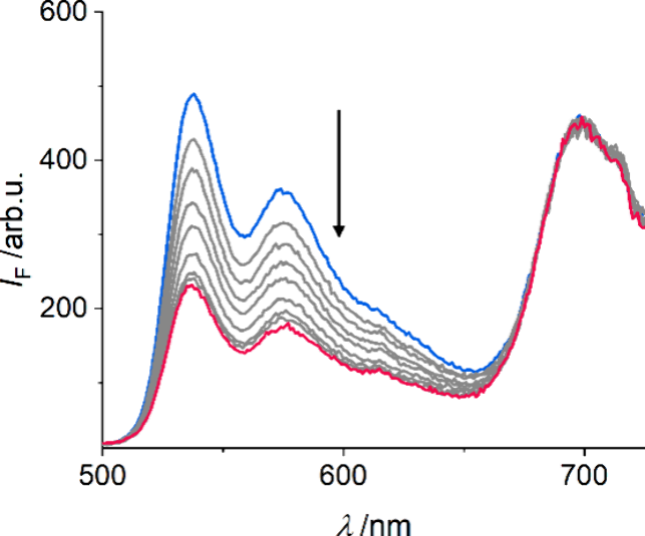
Fluorescence responses of **gMIP@rCS** upon gradual addition of FEX.TBA in MeCN (0–1.2 mM, start and end point spectra in blue and red), λ_exc_: 375 nm.

As depicted in Figure 7, the ratiometric probe showed well-resolved emission maxima at 538, 575, and 698 nm. With an increase in the concentration of FEX.TBA, the fluorescence intensity from **M**_**1**_ (538 and 575 nm) decreased steadily, echoing well the response of **M**_**1**_ in dilute solution. The fluorescence changes at 538 and 575 nm were similar upon addition of the analyte (Figure S17), further confirming that the intermediate emission at ca. 590 nm originates from species of **M**_**1**_ that show an identical binding behavior in the ground state.

Additionally, the fluorescence intensity from the **rCS** core (ca. 690 nm) remained constant, because of the absence of an interaction of the analyte with **I** in the core, allowing to use the **rCS** emission for internal referencing. Adequate limit of blank (LOB) and limit of detection (LOD) of 71 nM and 132 nM, respectively, were obtained for the MIP system, with limit of quantitation (LOQ) as low as 163 nM and a broad dynamic working range up to 1.2 mM (see Figure S18 and Section XXIII, Supporting Information for more details).

As can be deduced from Figure S18, the response of the particle probes is reversible, i.e., dilution after analyte addition leads to the expected signal change in the opposite direction, with referencing to the red fluorescence contributing to reliability and immediate visibility. If combined for instance with microfluidic assay approaches^11^ this system might be suitable for the detection of contaminated samples.^33,34^

Having established the sensitivity performance, the selectivity of ratiometric **gMIP@rCS** particles was further investigated. Two antibiotics containing carboxylate, phenyl, amino and/or hydroxyl groups, ampicillin (AMPI) and amoxicillin (AMOX), were first analyzed as potential competitors. In addition, AMPI and AMOX are significantly smaller than FEX, i.e., their molecular long axis being approximately 14 Å compared to approximately 21 Å for FEX, which gives a good indication of the discriminatory ability of the MIP.

**Figure 8:**
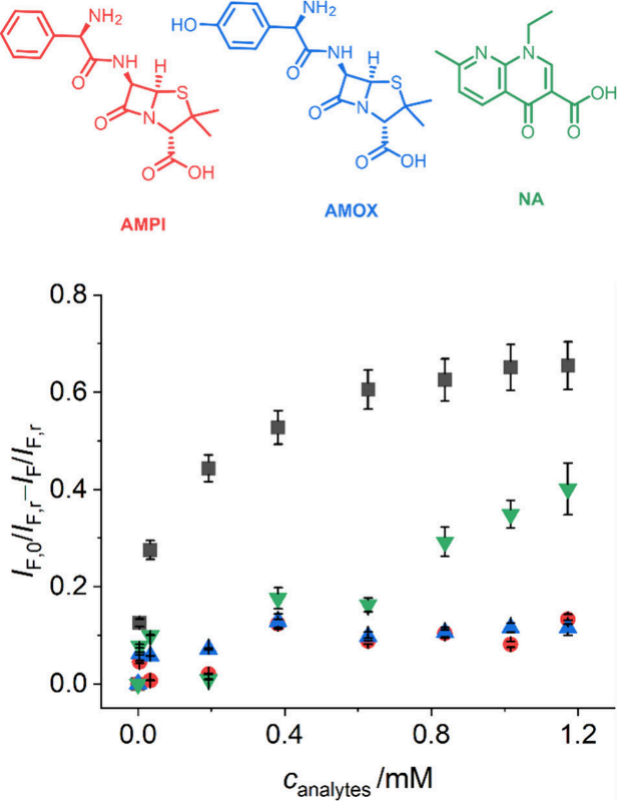
Top: Chemical structures of the potential competitors, ampicillin (AMPI), amoxicillin (AMOX) and nalidixic acid (NA), all of which were used as their TBA salts. Bottom: Ratiometric fluorescence response of **gMIP@rCS** toward FEX.TBA (black squares) and its competitors AMPI.TBA (red circles), AMOX.TBA (blue triangles), and NA.TBA (green triangles). *I*_F,0_ and *I*_F_ refer to the fluorescence intensity at 538 nm without and with analyte, and *I*_F,r_ denotes the fluorescence intensity at 690 nm.

As can be seen in Figures 7 and 8, whereas the addition of FEX.TBA resulted in a 65% decrease in the ratio of fluorescence intensity at 538 nm (*I*_F_) relative to 690 nm (*I*_F, r_), upon the addition of AMPI and AMOX, as their TBA salts, the ratios of the two emissions were only initially reduced by ca. 7%—most likely because of interaction with **M**_**1**_ units residing close to the outer surface of the shell and not in well-formed cavities—and then remained virtually unchanged.

Furthermore, as is shown in Figures S19 and S21, the fluorescence quenching efficiency of these potential competitors is ca. 10-fold weaker for **gMIP@rCS** compared to neat **M**_**1**_, stressing how the effective molecular imprinting of FEX.TBA significantly improves the selectivity of the **gMIP@rCS** probe.

In addition, considering the potential interference of the charge density on the carboxylate group of an analyte molecule toward its binding with the indicator monomer **M**_**1**_, we examined another carboxylate-containing drug with a higher electron density in its carboxylate moiety yet a smaller overall size, nalidixic acid (NA, long axis of approximately 9 Å), also as its TBA salt.

Figures 8 and S20 reveal that the addition of NA.TBA led to a moderate reduction in the ratiometric fluorescence intensity, distinctly less as for FEX.TBA. In addition, Figure 8 shows that the moderate response toward NA.TBA is only seen at distinctly higher concentrations, further proving the formation of specific recognition cavities in the **gMIP@rCS** matrix for FEX.

The selective recognition ability of **gMIP@rCS** was quantified via the discrimination factor (DF), determined as the change of relative fluorescence intensity of **gMIP@rCS** against FEX.TBA to **gMIP@rCS** versus the competitors, resulting in DF = 6.1, 5.8, and 1.8 for AMPI.TBA, AMOX.TBA and NA.TBA, respectively. These results confirm that the present approach is promising for devising ratiometric fluorescence sensing systems on the basis of thin MIP shells on submicron particle cores.

## Conclusions

This paper presents a promising approach for the development of ratiometric fluorescent particle probes for the detection of small molecule analytes. It is a generic concept based on BODIPY dyes, exploiting their unique spectroscopic and photophysical properties and combining them with molecularly imprinted polymer recognition matrices and the robustness and ease of use of core–shell (sub)microparticles.

Specifically, a green-emitting BODIPY indicator monomer, capable of binding carboxylate-expressing molecules by hydrogen bonding, was integrated into a MIP layer so that the sensing layer, which is only a few nanometers thin, responds to the imprinted model analyte fexofenadine by a change in fluorescence. A red-emitting BODIPY dye was doped into polystyrene core particles to generate a stable reference signal, while a thin silica shell between the two polymer entities shields the core from environmental influences and prevents crosstalk between the dyes in the core and shell.

The system offers excellent modularity in terms of choice of wavelength ranges, target analytes/templates, particle size and encoding through the versatility of BODIPY, MIP, silane and polystyrene chemistry. As a final result, the MIP probes respond to FEX over a wide dynamic range (>4 orders of magnitude) down to submicromolar concentrations. This performance is comparable to current electrochemical and optical methods, offering significant potential for future sensor development (see Table S5, Supporting Information).^63−66^

Despite these achievements, improvements in sensitivity are required for (ultra)trace analysis, which may involve preconcentration steps—the classical application of bulk MIPs^67,68^—or biphasic extraction.^11,69,70^ However, the presented toolbox already offers a variety of possibilities for device integration, be it on well plates, in microfluidics or in lateral flow. For example, micron-sized particles could be used for single particle applications or submicron or nanosized particles for bulk applications, to finally take the step toward multiplexed detection of small molecules in simple but reliable and robust formats that would be required to provide point-of-need analytical solutions in many environmental monitoring, food analysis or drug detection scenarios.

## Supporting Information Available

Syntheses, characterization of dyes, particle probes; pretreatment of analytes; 3D and fluorescence quantum yield measurements; crystal data and structure refinement; absorption, fluorescence and NMR titrations; quantum chemical considerations; mechanistic studies on particle probes; determination of LOB and LOD and measurement uncertainties; comparison with other methods (PDF)
