# Supplementary material for: Ratiometric Molecularly Imprinted Particle Probes for Reliable Fluorescence Signaling of Carboxylate-Containing Molecules
Source: ACS Appl Mater Interfaces. 2024 Sep 4;16(37):49944–56. doi: 10.1021/acsami.4c09990 (PMC11420868; doi:10.1021/acsami.4c09990)
Supplement: Supplementary file 1 — am4c09990_si_001.pdf [file am4c09990_si_001.pdf]

# Supporting Information

# Ratiometric Molecularly Imprinted Particle Probes for Reliable Fluorescence Signaling of Carboxylate-Containing Molecules

Yijuan Sun, Kornelia Gawlitza, Virginia Valderrey, Biswajit Bhattacharya and Knut Rurack*

Bundesanstalt für Materialforschung und -prüfung (BAM)

Richard-Willstätter-Str. 11, 12489 Berlin, Germany

knut.rurack@bam.de

## Table of Contents

I. Measurement and Analysis of 3-Dimensional Fluorescence Decays ........ S3

II. Synthesis of *meso*-(4-Aminophenyl)-BODIPY **2** ........ S3

III. Synthesis of Red BODIPY Dye **I** ........ S4

IV. Preparation of Polystyrene Core Doped with **I** ........ S4

V. Preparation of Silica-Coated rPS (**rCS**) ........ S5

VI. Functionalization of **rCS** Particles ........ S5

VII. Pretreatment of Analytes ........ S6

VIII. Determination of Fluorescence Quantum Yields ........ S6

IX. Crystallographic Information Crystal Data and Structure Refinement ........ S7

X. Absorption Titration Spectra ........ S8

XI. Quantum Chemical Considerations ........ S9

XII. Fluorescence Responses of $\mathbf{M_1}$ to AcO.TBA in THF and Toluene ........ S13

XIII. Evaluation of Binding Constants via Fluorescence Titrations ........ S14

XIV. $^{1}H$ NMR Titrations ........ S15

XV. Absorption Titration Spectra of $\mathbf{M_1}$ against FEX.TBA ........ S15

XVI. Zeta Potential and TGA Profiles ........ S16

XVII. Absorption and Fluorescence Spectra of Pre-Polymerization Mixtures ........ S17

XVIII. Optimizing the Amount of Initiator ........ S18

XIX. Optimization of Polymerisation Time ........ S19

XX. Studies on the Origin of the Unusually Structured Emission of **gMIP@rCS** ........ S21

XXI. Reproduction of **gMIP@rCS** ........ S25

XXII. Fluorescence Changes of **gMIP@rCS** ........ S25

XXIII. Determination of LOB and LOD / Demonstration of Reversibility ........ S26

XXIV. Fluorescence Responses of **gMIP@rCS** to Competitors ........ S27

XXV. Fluorescence Responses of $\mathbf{M_1}$ to Competitors ........ S28

XXVI. Comparison with Other Methods for the Detection of Fexofenadine ........ S29

XXVII. Calculation of Measurement Uncertainties ........ S31

XXVIII. $^{1}H$ and $^{13}C$ NMR Spectra of BODIPYs ........ S33

XXIX. Detailed Results of Quantum Chemical Calculations Following Various Refs[8-13] ........ S39

XXX. References ........ S42

## I. Measurement and Analysis of 3-Dimensional Fluorescence Decays

The fluorescence lifetime profiles were analyzed with the High-Performance Digital Temporal Analyzer (HPD-TA) software package including the TA-fit with the global deconvolution fitting module (Hamamatsu). The three-dimensional camera images were first grouped into regions of 20 nm (515–535, 535–555, 555–575 and 575–595 nm) and fitted according to the relevant decay model. These 20-nm decay traces were then taken together for the global analysis. The goodness-of-fit of the single decay regions and the global analysis were judged by reduced chi-squared ($\chi_R^2$) and the autocorrelation function $C$(j) of the residuals, varying between $1.0 < \chi_R^2 < 1.9$, the rather high $\chi_R^2$ results stemming from the traces recorded at >570 nm that were of considerably low intensity.

## II. Synthesis of *meso*-(4-Aminophenyl)-BODIPY 2

***meso*-(4-Nitrophenyl)-BODIPY 3**: 2,4-Dimethyl-3-ethylpyrrole (0.54 mL, 4 mmol) was added to a solution of 4-nitrobenzoyl chloride (370 mg, 2 mmol) in anhydrous $CH_2Cl_2$ (45 mL) under an argon atmosphere. After stirring at room temperature for 4 d, triethylamine (1.67 mL, 12 mmol) was added at 0 °C and stirred for 10 min, followed by the addition of boron trifluoride-diethyl etherate (1.98 mL, 16 mmol) to the solution. The reaction solution was stirred for 12 h at room temperature, after which the reaction was quenched with saturated aqueous $NaHCO_3$ and extracted with $CH_2Cl_2$. The organic layer was collected and dried over $Na_2SO_4$, followed by concentration under reduced pressure. The crude product was purified by column chromatography on silica using EtOAc/cyclohexane (1/5, v/v) as eluent to afford *meso*-(4-nitrophenyl)-BODIPY **3** as red crystals (434 mg, 51%). $^1$H NMR (400 MHz, $CDCl_3$): δ (ppm) = 8.39 (d, $J$ = 8.7 Hz, 2H), 7.55 (d, $J$ = 8.7 Hz, 2H), 2.54 (s, 6H), 2.33 (q, $J$ = 7.6 Hz, 4H), 1.26 (s, 6H), 1.00 (t, $J$ = 7.6 Hz, 6H). $^{13}$C NMR (101 MHz, $CDCl_3$): δ (ppm) = 155.13, 148.40, 143.03, 137.81, 136.96, 133.66, 130.14, 130.08, 124.39, 17.21, 14.69, 12.74, 12.15. HRMS-ESI: m/z calculated for $C_{23}H_{26}BF_2N_3O_2$ $[M]^+$: 425.2086, found: 425.2046.

***meso*-(4-Aminophenyl)-BODIPY 2**: **3** (425 mg, 1 mmol) and 10% Pd/C (53 mg, 50 µmol) were dissolved in a degassed mixture of $CH_2Cl_2$/MeOH (20 mL/20 mL). The solution was saturated with hydrogen gas and stirred at room temperature for 15 h. The Pd/C was removed by filtration through celite and flushed with $CH_2Cl_2$. The solvent was evaporated to *meso*-(4-aminophenyl)-BODIPY **2**

as an orange powder (375 mg, 95%). $^{1}$H NMR (400 MHz, $CDCl_3$): δ (ppm) = 7.02 (d, *J* = 8.6 Hz, 2H), 6.79 (d, *J* = 8.6 Hz, 2H), 3.82 (s, 2H), 2.52 (s, 6H), 2.33 (q, *J* = 7.6 Hz, 4H), 1.40 (s, 6H), 1.00 (t, *J* = 7.6 Hz, 6H). $^{13}$C NMR (101 MHz, $CDCl_3$): δ (ppm) = 153.38, 146.60, 141.11, 138.64, 132.64, 131.49, 129.42, 126.04, 115.75, 17.23, 14.78, 12.60, 12.07. HRMS-ESI: m/z calculated for $C_{23}H_{29}BF_2N_3$ $[M+H]^+$: 396.2356, found: 396.2423.

## III. Synthesis of Red BODIPY Dye I

The synthesis of red BODIPY dye **I** was performed as described in previous work.[1] Firstly, pentafluorobenzaldehyde (98 mg, 0.5 mmol) and 2,4-dimethylpyrrole (105 mg, 1.1 mmol) were dissolved in anhydrous $CH_2Cl_2$ (15 mL), followed by slow addition of a few drops of trifluoroacetic acid. The reaction solution was stirred at room temperature for ca. 4 h until the aldehyde was completely consumed, whereupon 2,3-dichloro-5,6-dicyano-1,4-benzoquinone (136 mg, 0.6 mmol) was added. After stirring the mixture at room temperature for 1 h, triethylamine (2 mL, 14 mmol) was added to the cooled mixture, and stirring was continued for 10 min before boron trifluoride-diethyl etherate (2.5 mL, 20 mmol) was subsequently added dropwise. The reaction mixture was further stirred at room temperature for 2 h. The solvent was evaporated under vacuum, and the product was purified by column chromatography on silica gel using $CH_2Cl_2$/cyclohexane (1/6, v/v) as eluent to give *meso*-pentafluorophenyl-BODIPY **II** as an orange solid (54 mg, 26%). Subsequently, **II** (50 mg, 0.12 mmol) and *p*-anisaldehyde (36 mg, 0.27 mmol) were dissolved in dry toluene (12 mL), after which piperidine (0.6 mL, 6 mmol), a few drops of glacial acetic acid and activated molecular sieves (4 Å) were added. The mixture was refluxed overnight before cooling to room temperature. Afterwards, toluene was evaporated under reduced pressure. The resulting crude residue was purified through a silica column using $CH_2Cl_2$/cyclohexane (1/5, v/v) as eluent and recrystallized from $CH_2Cl_2$/hexane to afford **I** as a brown solid (44 mg, 56%). $^{1}$H NMR (400 MHz, $CDCl_3$): δ (ppm) = 7.63–7.58 (m, 6H), 7.29 (s, 1H), 7.25 (s, 1H), 6.96–6.92 (m, 4H), 6.68 (s, 2H), 3.86 (s, 6H), 1.68 (s, 6H). $^{13}$C NMR (101 MHz, $CDCl_3$): δ (ppm) = 160.95, 154.28, 139.86, 137.48, 133.00, 129.48, 118.63, 118.32, 117.07, 114.53, 55.55, 13.93.

## IV. Preparation of Polystyrene Core Doped with I

**Polystyrene Core (PS):** The synthesis of polystyrene (PS) particles was performed according to a published protocol with some modifications.[2] The purchased styrene was pretreated with a column

packed with basic alumina to remove the inhibitor 4-*tert*-butylcatechol prior to polymerization. Inhibitor-free styrene (2.2 mL, 19.20 mmol) was added to degassed Milli-Q water (88 mL) and flushed with argon while stirring for 15 min to ensure that the styrene was well dissolved in the aqueous solution. Meanwhile, AIBA (42 mg, 0.15 mmol) was dissolved in Milli-Q water (10 mL) and degassed with argon for 10 min. To start the polymerization, the AIBA solution was added to the styrene dispersion. The reaction was proceeded at 55 °C under continuous mixing for 17 h. The resulting suspension was cooled down to room temperature and stored at 4 °C.

**Doping of PS Core Particles with I (rPS):** Based on a previously published procedure,[3] THF (250 μL) was added to a suspension of the above obtained positively charged PS particles (28 mL), immediately seated on a rotator and mixed at 40 rpm for 1 h at room temperature. 2 mL of **I** (2 mM) in THF was subsequently added to the solution. After rotating for another 2 h at room temperature, the red BODIPY-doped particles (**rPS**) were collected and washed twice with Milli-Q water (30 mL) by centrifugation at 9140 ×*g* for 30 min., then dried in a vacuum for 5 h.

## V. Preparation of Silica-Coated rPS (rCS)

Silica shells were decorated onto the **rPS** core particles by suspending the PS beads (175 mg) uniformly in a mixture of isopropanol (87 mL) and Milli-Q water (3 mL), following a previously published approach but using isopropanol instead of ethanol as a solvent.[4] Thereafter, ammonia solution (32%, 1.6 mL) and TEOS (1.6 mL, 7.17 mmol) were sequentially added to the suspension. The reaction was left to proceed for 15 h at 38 °C under constant mixing. The resulting particles were collected by centrifugation and washed twice with Milli-Q water and ethanol (9140 ×*g*, 10 min.), after which the silica-coated **rPS** core-shell (**rCS**) particles were dried in a vacuum.

## VI. Functionalization of rCS Particles

**APTES Modification:** Following our earlier work,[5] 1.2 M HCl (5 mL) was added to homogeneously dispersed **rCS** particles (420 mg) in absolute ethanol (10 mL), and the dispersion was ultrasound-treated for 15 min. The particles were recovered by centrifugation (9140 ×*g*, 15 min) and washed twice with absolute ethanol (30 mL). Afterwards, the obtained particles were re-dispersed in absolute ethanol (8 mL) and transferred to a round-bottomed flask, followed by the addition of APTES (0.42 mL, 1.79 mmol) to the suspension, which was stirred at 40 °C for 24 h.

After that, the particles were collected and rinsed three times with absolute ethanol (30 mL) by centrifugation (9140 ×*g*, 15 min.). The APTES-modified particles (**a@rCS**) were dried under vacuum overnight.

## VII. Pretreatment of Analytes

The commercially available analyte was treated by evenly dispersing the corresponding molecule (0.15 mmol) in acetonitrile (4 mL), after which tetrabutylammonium hydroxide solution in methanol (1M) was added in an equimolar proportion. The solution was sonicated for 15 min and mounted onto a rotator for further mixing at 40 rpm for 1 h. The mixture was concentrated in a vacuum concentrator at 200 mbar for 20 min and kept at 0 mbar for another 1 h. The mixture was then dried under vacuum for 12 h.

## VIII. Determination of Fluorescence Quantum Yields

The fluorescence quantum yield of $\mathbf{M_1}$ and $\mathbf{M_2}$ were determined relative to rhodamine 6G in ethanol ($\Phi_f = 0.91$, $\lambda_{exc}$: 488 nm)[6] and $\Phi_f$ of **I** in MeCN was determined to $\Phi_f = 0.641 \pm 0.02$ by comparison with oxazine 1 in ethanol ($\Phi_f = 0.14$).[7]

## IX. Crystallographic Information Crystal Data and Structure Refinement

**Table S1.** Crystallographic information of **$M_1$** and **$M_2$**.

| **Compounds** | **$M_1$** | **$M_2$** |
|---|---|---|
| Formula | $C_{60}H_{74}B_2F_4N_8O_4S_2$ | $C_{31}H_{39}BCl_2F_2N_4O_3$ |
| Formula weight | 1133.01 | 635.37 |
| T/K | 150 | 150 |
| Crystal system | Triclinic | Orthorhombic |
| Space group | $P\bar{1}$ | $Pna2_1$ |
| a/Å | 11.1651(4) | 9.0373(3) |
| b/Å | 16.4903(7) | 12.7258(5) |
| c/Å | 17.3496(7) | 27.9730(10) |
| α/° | 71.126(2) | 90 |
| b/° | 84.895(2) | 90 |
| γ/° | 76.724(2) | 90 |
| Volume/Å $^3$ | 2941.4(2) | 3217.1(2) |
| *Z* | 2 | 4 |
| ρ, Mg.cm$^{-3}$ | 1.279 | 1.312 |
| μ /mm$^{-1}$ | 0.157 | 0.251 |
| F(000) | 1200.0 | 1336.0 |
| 2θ range | 4.846-53.484 | 5.528-53.752 |
| Reflections Collected | 137579 | 65235 |
| Independent Reflections | 11969 | 6758 |
| $R_{int}$ | 0.0372 | 0.0577 |
| GOF | 1.036 | 1.060 |
| R1 ($I > 2\sigma(I)$) | 0.0517 | 0.0495 |
| $wR2(I > 2\sigma(I))$ | 0.1319 | 0.1299 |
| CCDC Number | 2290578 | 2290577 |

## X. Absorption Titration Spectra

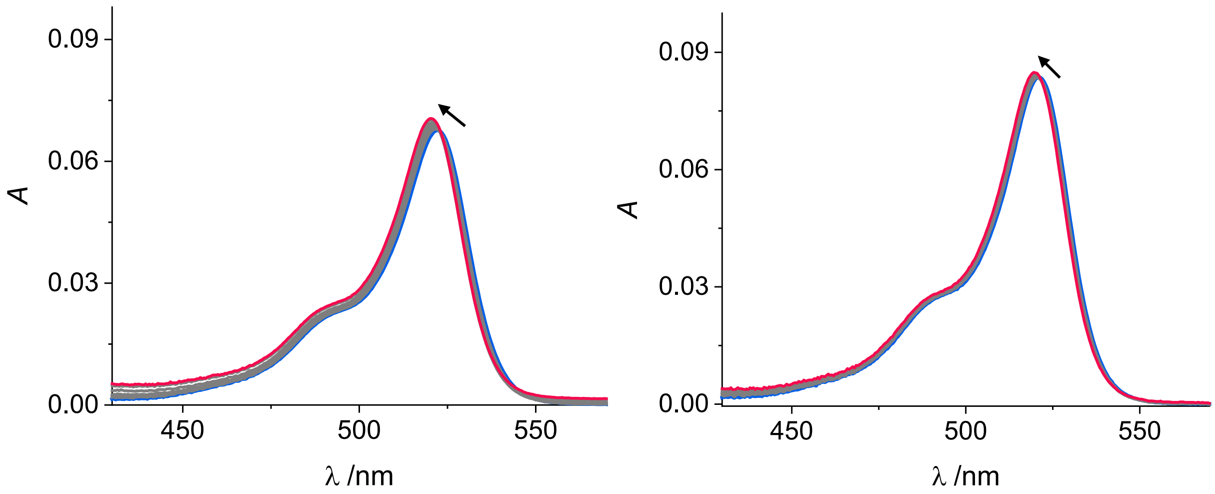


**Figure S1.** Absorption titration spectra of $\mathbf{M_1}$ (left) and $\mathbf{M_2}$ (right), $c_{\mathbf{Mx}}$ = 1 μM, with increasing concentrations of AcO.TBA in MeCN (0–190 μM for $\mathbf{M_1}$, 0–150 μM for $\mathbf{M_2}$; start and end point spectra shown in blue—barely visible due to strong overlap—and red).

## XI. Quantum Chemical Considerations

The consistent and correct theoretical reproduction of experimental absorption spectra and maxima is still a challenge for polymethine dyes, including BODIPY dyes, when employing the perhaps today most widely used quantum chemical approaches that offer the best compromise between sophistication and computing time for such molecular systems, i.e., density functional theory (DFT) and time-dependent density functional theory (TD-DFT) methods.[8-13]

Generally, the choice of the functional was found to have a more significant impact than the basis set, the latter covering the whole range from 6-31G to 6-311++G(d,p). Concerning the functionals, the CAM-B3LYP and the M06-2X were frequently found to yield the best correlations with experimental data (after application of appropriate correction factors), while B3LYP was found to be inferior.[8, 10, 13]

In our case, the challenge was not only to reproduce the behavior of the gross neutral polymethinic indicator monomer **M1**, but in the next step to assess mechanistically the influence of binding to the acetate anion or, more realistically,[14] the binding of $AcO^-$ in the presence of the $TBA^+$ counterion.

What we consistently observed in the experiments were small hypsochromic and hyperchromic shifts in absorption, together with a significant quenching of the fluorescence, irrespective of the solvent polarity, i.e., by a factor of ca. 10 in highly polar MeCN and by a factor of ca. 2 in non-polar toluene. However, no matter whether we took the CAM-B3LYP or the M06-2X functional for both, geometry optimization and vertical transitions or the B3LYP for geometry optimization and the other two for the transitions, and irrespective of the basis set, theory always reproduced the binding-induced hypsochromic shift yet yielded a small hypo- instead of a hyperchromic shift for the BODIPY-localized transitions in the ternary complex.

Moreover, the calculations did not reveal any immediately obvious explanation for low-lying and largely forbidden states in the complexes which are often associated with the observed quenching of such systems via excited-state charge (CT) or (photoinduced) electron transfer (ET),[15] see Section XXIX at the end of this document.

Across 12 different theoretical approaches, the average spectral shift was calculated to $-2.0 \pm 0.5$ nm, the average change in oscillator strength to $-0.005 \pm 0.012$ and the average change in dipole

moment between ground and excited state to +1.4 ± 0.4 D for the lowest transition between free and complexed **M1**, i.e., no indication of highly dipolar CT or ET states were found (Section XXIX; illustration by a representative example in Table S2 and Figure S2).

**Table S2.** Calculated properties of the vertical excitation of the most stable energy-minimized ground-state geometries of **M1** and **M1**–AcO.TBA by TD-DFT; B3LYP functional with 6-31+G(d) basis set for geometry optimization and CAM-B3YLP functional with 6-311++G(d,p) basis set for vertical excitations.

| | $\lambda_{S_n \leftarrow S_0}(n)$ /nm [a] | $f$ [b] | $\Delta\mu_{S_n - S_0}$ /D [c] | fragment [d] | Orbitals (coefficients) [e] |
|---|---|---|---|---|---|
| M1 | 436.7 (1) | 0.585 | −1.5 | BDP | HOMO–LUMO (0.697) |
| | 336.0 (2) | 0.075 | −0.8 | BDP | HOMO−3–LUMO (0.695) |
| | 321.4 (3) | 0.001 | −0.3 | PhThUr | HOMO−1–LUMO+2 (0.492), HOMO−1–LUMO+1 (−0.336), etc |
| | 313.6 (4) | 0.064 | 0.0 | BDP | HOMO−4–LUMO (0.688) |
| M1–AcO.TBA | 434.4 (1) | 0.563 | −0.2 | BDP | HOMO–LUMO (0.697) |
| | 353.5 (2) | 0.083 | +13.7 | PhThUr–BDP | HOMO−2–LUMO (0.579), HOMO−3–LUMO (−0.358), etc |
| | 334.1 (3) | 0.068 | −2.4 | BDP | HOMO−4–LUMO (0.695) |
| | 311.8 (4) | 0.001 | +5.8 | PhThUr–BDP, PhThUr | HOMO−1–LUMO (0.382), HOMO−1–LUMO+7 (0.304), etc |

[*a*] Wavelength of the transition, calculated for a medium polar solvent such as chloroform according to a polarizable continuum model (IEFPCM). [*b*] Oscillator strength of the transition. [*c*] Dipole moment difference between ground ($\mu_0$) and respective excited ($\mu_n$) state. [*d*] Fragment on which the transition is mainly localized. [*e*] MOs involved in the transitions.

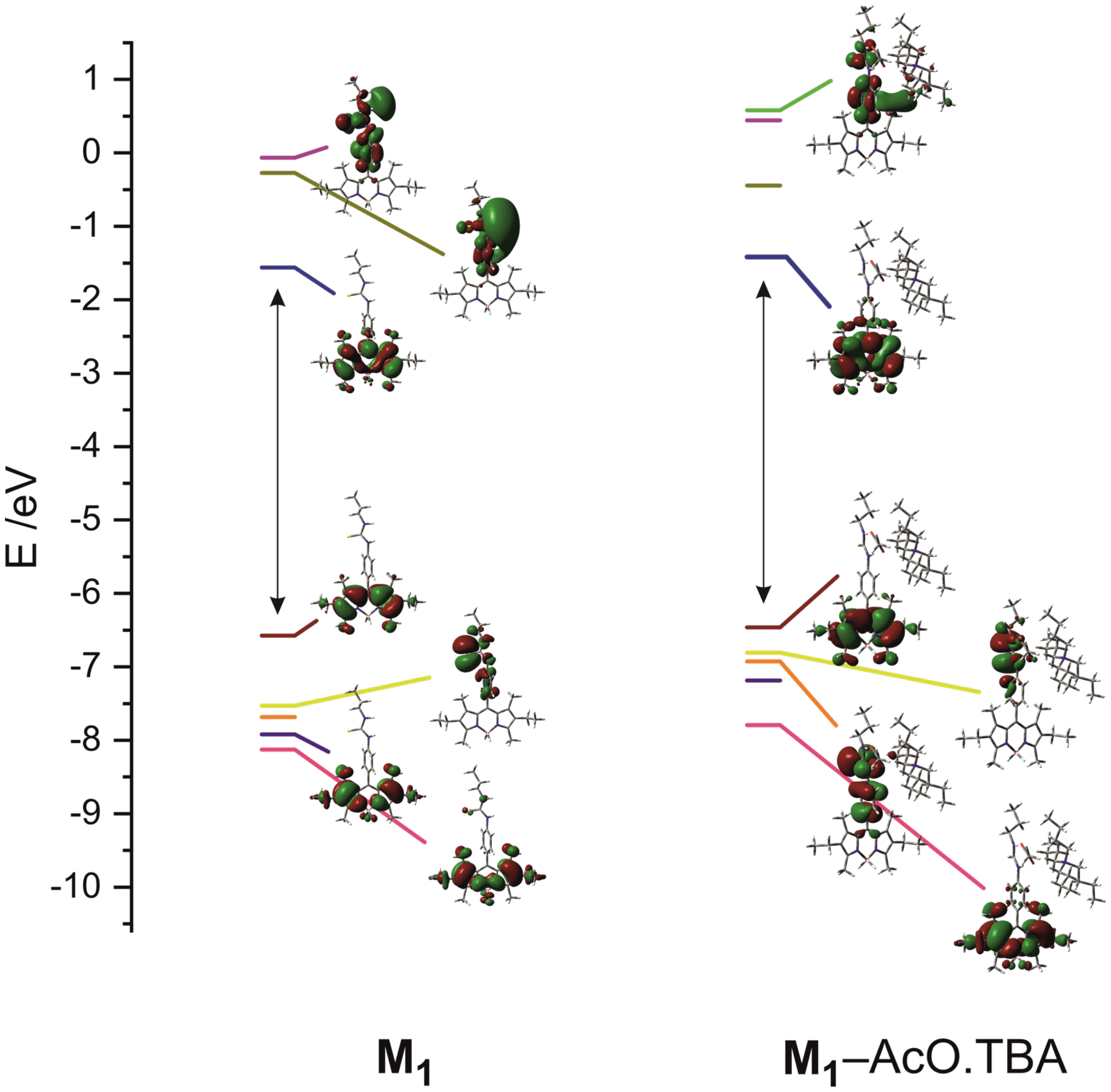


**Figure S2.** Energy levels of relevant frontier molecular orbitals HOMO (to HOMO−4) and LUMO (to LUMO+3) of **M**$_1$ and its AcO.TBA complex; localization of MOs on molecular fragments is only shown for relevant MOs listed in Table S2. B3LYP functional with 6-31+G(d) basis set for geometry optimization and CAM-B3YLP functional with 6-311++G(d,p) basis sets for vertical excitations.

A closer inspection of the four lowest transitions of **M**$_1$ in Table S2 reveals that no CT- or ET-active transition between the two orthogonally oriented molecular fragments is located within 1.1 eV of the $S_1$ transition, rendering any quenching interaction highly unlikely and agreeing well with the high fluorescence quantum yields found for **M**$_1$ in all the solvents investigated (Table 1).

For **M1**–AcO.TBA, the situation looks different. Although the lowest lying transition is still BODIPY-centered, see Table S2 and Figure S2, the $S_2$ transition is a classical ET transition, involving HOMO−2 and HOMO−3 centered on the *meso*-phenyl-thiourea fragment and LUMO centered on the BODIPY fragment and being associated with rather small oscillator strengths and a pronounced change in dipole moment (Table S2, Figure S2).

The states were thus analysed in more detail with respect to the question if stabilization by solvents of different polarities can lead to a state reversal so that the quenching ET state becomes the energetically most favored one (Table S3).

**Table S3.** Calculated electronic properties of the BODIPY-localized ($^1$LE) and the $^1$ET states of **M1**–AcO.TBA using the results of the DFT/TD-DFT calculations shown in Table S2.

| | $S_n$ | solvent | $\Delta E_{S_n \leftarrow S_0}(\mathrm{sol})$ [a] /eV | $\Delta E_{S_n \leftarrow S_0}(gas)$ [b] /eV | $\Delta\mu_{S_n - S_0}$ [c] /D | $\mu_{S_n}$ [d] /D | $f$ [e] | $\Delta E_{S_n - T_n}$ [f] /eV |
|---|---|---|---|---|---|---|---|---|
| $^1$LE | $S_1$ | MeCN | 2.86 | 2.85 | −0.2 | +15.0 | 0.083 | 1.63 |
| $^1$ET | $S_2$ | MeCN | 2.73 | 3.51 | +13.7 | +28.9 | 0.563 | 0.12 |
| $^1$LE | $S_1$ | THF | 2.86 | 2.85 | −0.2 | +15.0 | 0.083 | 1.63 |
| $^1$ET | $S_2$ | THF | 2.82 | 3.51 | +13.7 | +28.9 | 0.563 | 0.12 |
| $^1$LE | $S_1$ | Toluene | 2.86 | 2.85 | −0.2 | +15.0 | 0.083 | 1.63 |
| $^1$ET | $S_2$ | toluene | 3.02 | 3.51 | +13.7 | +28.9 | 0.563 | 0.12 |

[a] Calculated according to $\Delta E(sol) = \Delta E(gas) - \frac{1}{a_O{}^3}\frac{\epsilon_S - 1}{2\epsilon_S + 1}\left(\mu_{S_n}{}^2 - \mu_{S_n}\mu_{S_0}\right) - \frac{1}{a_O{}^3}\frac{n^2-1}{2n^2+1}\left(\mu_{S_n}\mu_{S_0} - \mu_{S_0}{}^2\right)$,[16, 17] with $\epsilon_S$ = 35.94, 7.58 and 2.38 for MeCN, THF and toluene,[18] n = 1.344, 1.407 and 1.497 for MeCN, THF and toluene;[18] $a_O$ = 6.64 Å (for emissive species **M1**), $\mu_{S_0}$ and $\mu_{S_n}$ obtained from DFT/TD-DFT calculations. [b] Energy of transition as obtained from TD-DFT calculations of the complexes in the gas phase, see Table S2. [c] Dipole moment difference between ground ($\mu_{S_0}$) and respective excited ($\mu_{S_n}$) state. [d] Dipole moment of excited state ($\mu_{S_n}$). [e] Oscillator strength of the transition. [f] Energy gap between respective singlet and triplet states.

The results of this analysis indeed reveal that especially polar solvents should stabilize the ET state in **M1**–AcO.TBA to such a degree that quenching occurs. For instance, whereas in the gas phase the energy gap for the $^1$ET state is higher than that for the BODIPY-localized $^1$LE state, $\Delta E_{S_n - S_0}$=

3.51 eV vs. 2.85 eV, this order is reversed in THF (2.82 vs. 2.85 eV) and especially MeCN (2.73 vs. 2.85 eV, Table S3).

Although the theoretical analysis predicts this effect only for MeCN and THF, but not for toluene, the latter mismatch with experiment, i.e., quenching in all three solvents, is attributed to the simplification of the system in the theoretical studies. The trend of quenching, however, MeCN > THF > toluene is well reflected by the results in Table S3 and Figure S3.

## XII. Fluorescence Responses of $M_1$ to AcO.TBA in THF and Toluene

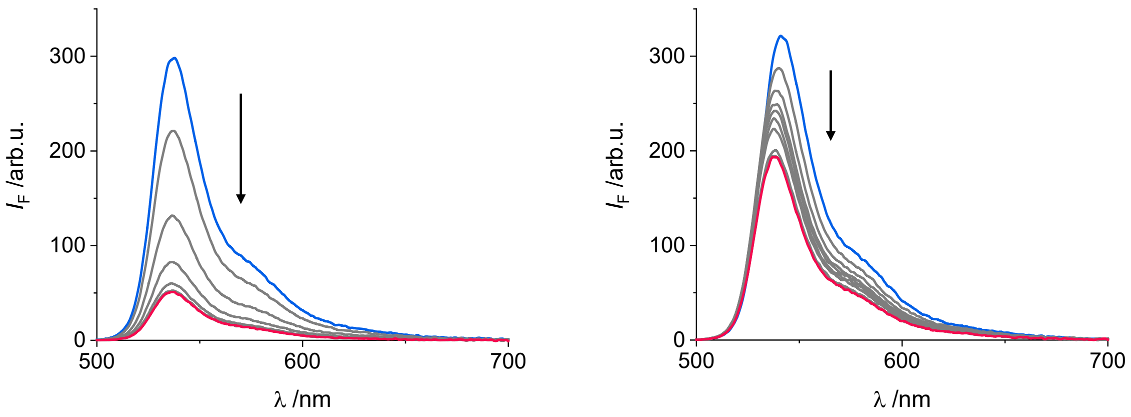


**Figure S3.** Fluorescence responses of $\mathbf{M_1}$ ($c$ = 1 μM) upon addition of AcO.TBA in THF (left, 0–6 μM, 83% fluorescence quenching; start and end point spectra shown in blue and red) and in toluene (right, 0–200 μM, 42% fluorescence quenching; start and end point spectra shown in blue and red); $\lambda_{exc}$: 490 nm.

## XIII. Evaluation of Binding Constants via Fluorescence Titrations

To assess the binding affinity of indicators **M1** and **M2** for AcO.TBA, **M1** and **M2** were titrated with analyte stock solutions corresponding to 0–190 μM AcO.TBA for **M1** and 0–150 μM for **M2** in acetonitrile until signal saturation was reached. The titration data were fitted with BindFit software to a 1:1 (host:guest) nonlinear binding model.[19]

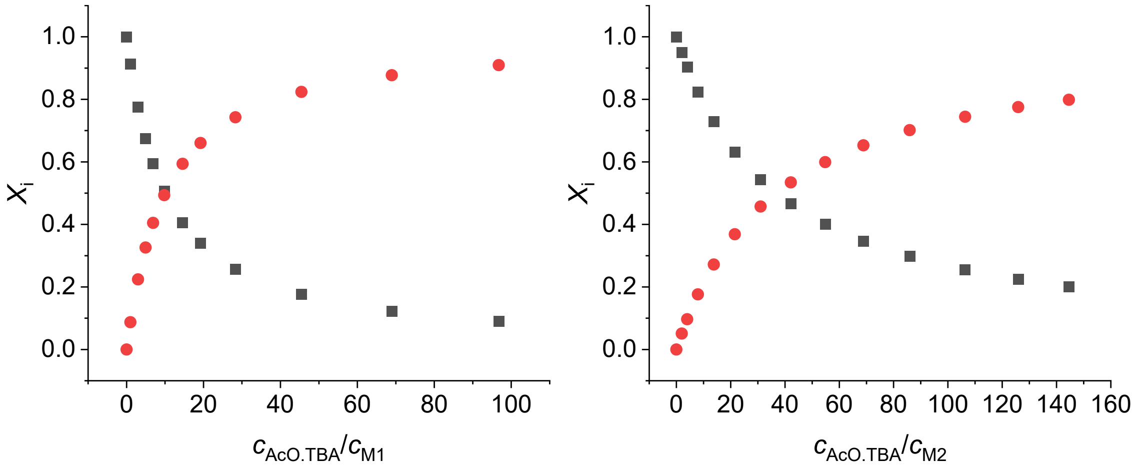


**Figure S4.** Variations in molar fractions ($X_i$) of free **M1** (left, black squares), **M2** (right, black squares) and their carboxylate complexes (red circles) with increasing concentrations of AcO.TBA during titrations in MeCN.

## XIV. $^1$H NMR Titrations

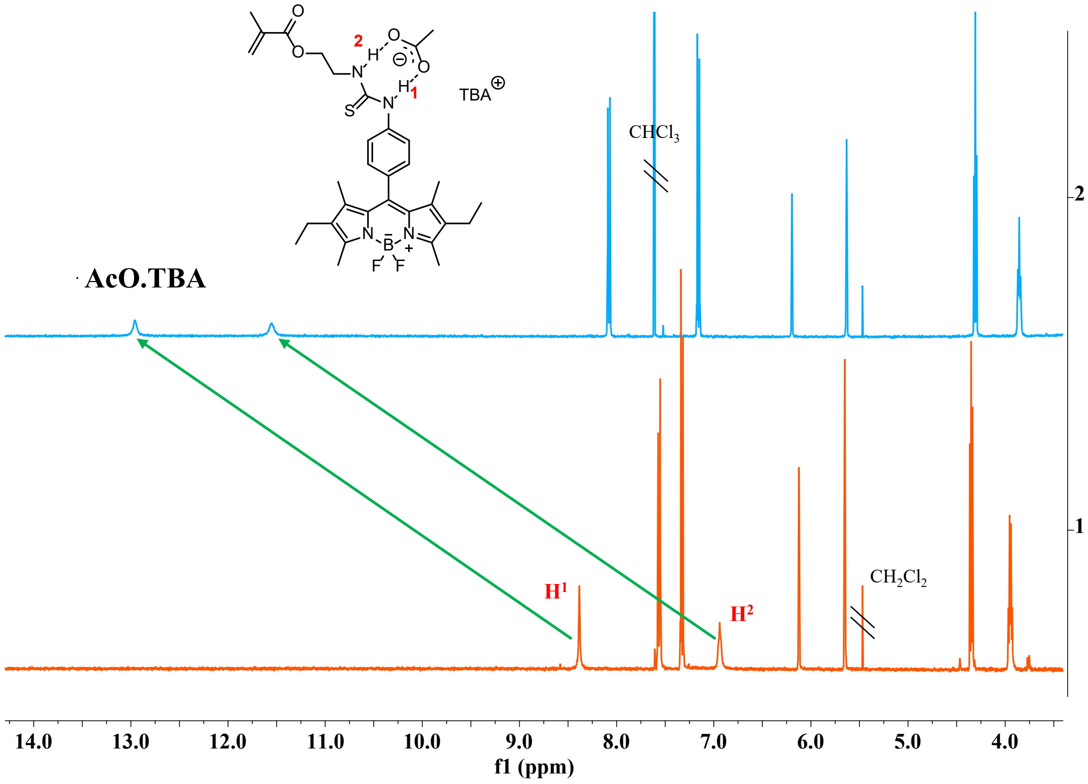


**Fig. S5.** Partial $^1$H NMR titration of $\mathbf{M_1}$ with 2 equiv. of AcO.TBA in $CD_3CN$.

## XV. Absorption Titration Spectra of $\mathbf{M_1}$ against FEX.TBA

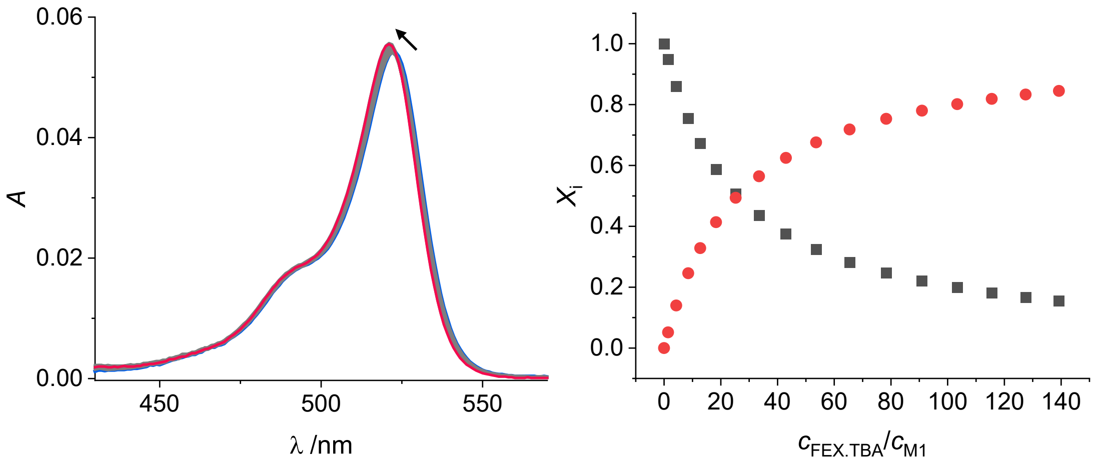


**Figure S6.** Left: Absorption spectra of indicator monomer $\mathbf{M_1}$ ($c$ = 0.7 μM) upon addition of FEX.TBA in MeCN (0–97 μM; end point spectrum shown in red). Right: Variations in molar fractions ($X_i$) of free $\mathbf{M_1}$ (black squares) and its FEX complex (red circles) with increasing concentrations of FEX.TBA during a titration.

## XVI. Zeta Potential and TGA Profiles

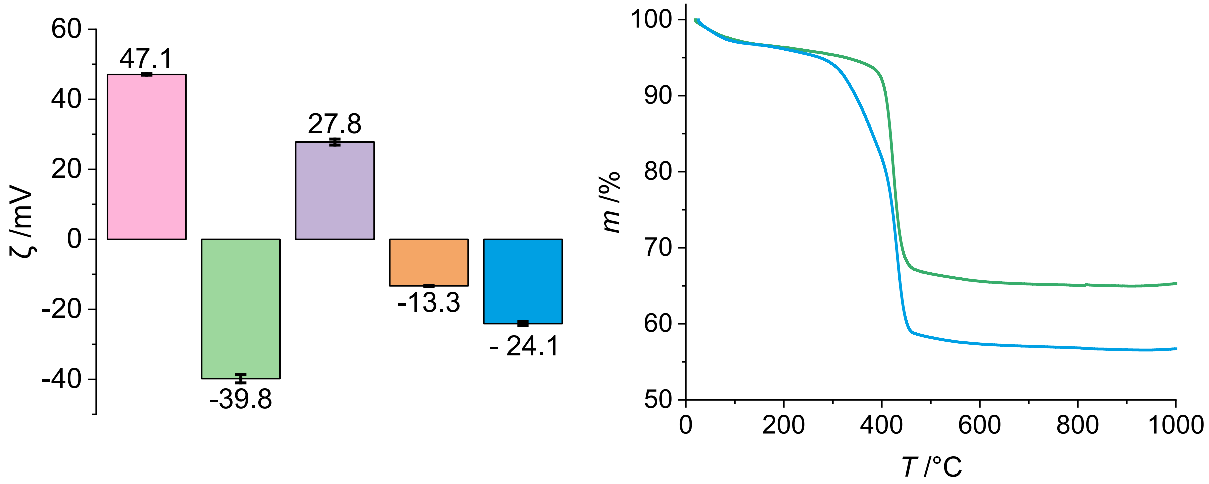


**Figure S7.** Left: Zeta potential of PS beads (pink), **rCS** particles (green), APTES-modified particles **a@rCS** (purple), RAFT-functionalized particles **raft@rCS** (orange) and **gMIP@rCS** particles (blue). Right: TGA curves of **rCS** particles (green) and **gMIP@rCS** particles (blue). The curves for RAFT-functionalized **rCS** and **a@rCS** are virtually identical to that of **rCS**.

## XVII. Absorption and Fluorescence Spectra of Pre-Polymerization Mixtures

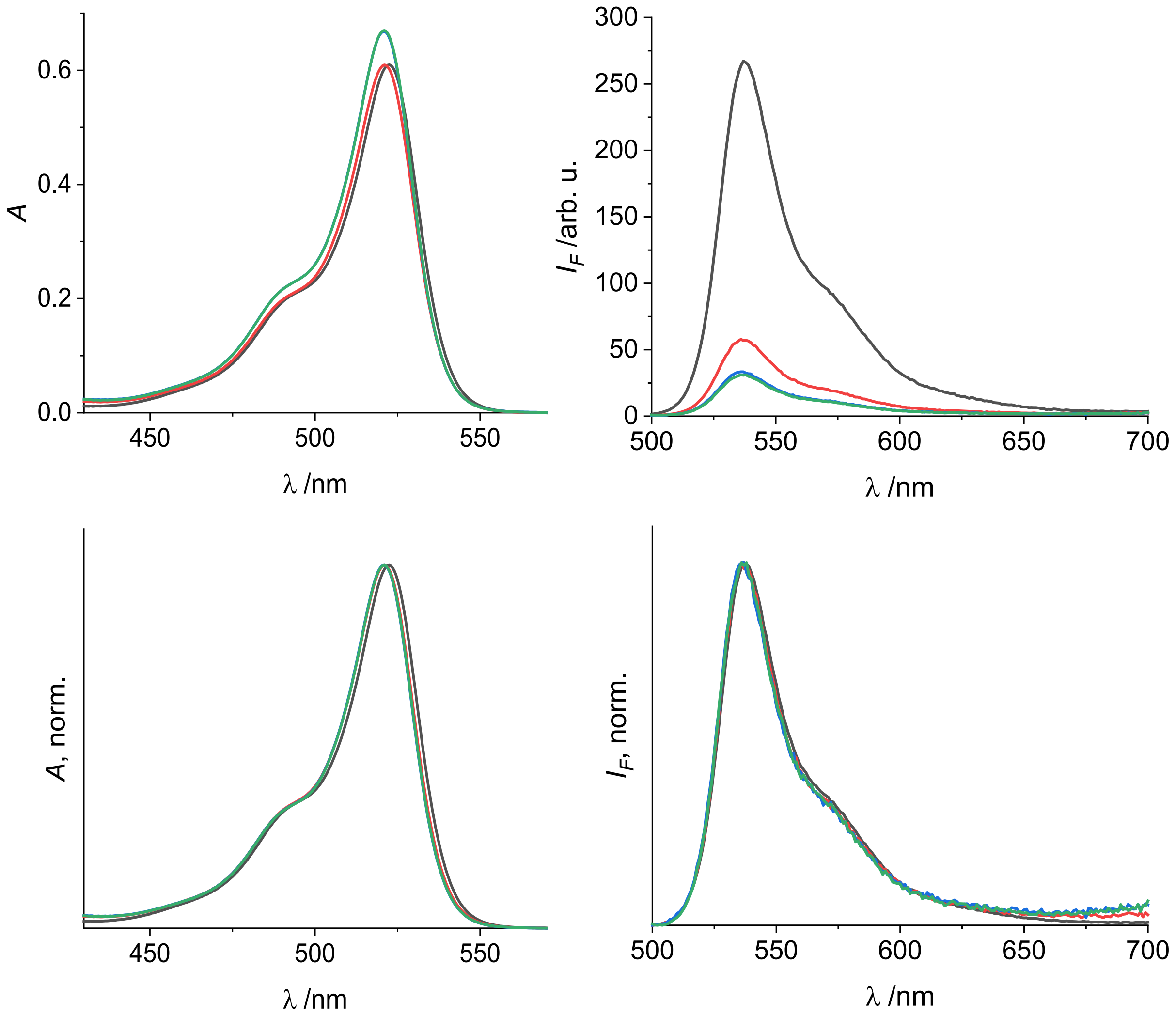


**Figure S8.** Absorption (top left), normalized absorption (bottom left), fluorescence (top right) and normalized fluorescence (bottom right) spectra of prepolymerization mixtures for the synthesis of **gMIP@rCS** particles in MeCN, employing microcuvettes with a pathlength of 100 μm (and in front-face geometry for fluorescence measurements). $\mathbf{M_1}$ (black line), $\mathbf{M_1}$ + 1 equiv. FEX.TBA (red line), $\mathbf{M_1}$ + 2 equiv. FEX.TBA (blue line) and $\mathbf{M_1}$ + 2 equiv. FEX.TBA + BMA + EGMDA (green line); $c_{\mathbf{M1}}$ = 0.75 mM.

## XVIII. Optimizing the Amount of Initiator

The ratio of the RAFT agent to initiator-derived radicals is a critical parameter and cannot be arbitrarily selected. Here, the RAFT-grafted core particles were designed to incorporate pentafluorophenyl-substituted BODIPY dyes, each molecule containing seven fluorine atoms. This composition posed challenges in accurately determining the sulfur content in the RAFT-grafted particles using elemental analysis, which is the most suitable direct quantification method as S atoms are unique to the RAFT agent in our system.

It is proposed that during high-temperature combustion, fluorine may react with sulfur, preventing the conversion of S into $SO_2$, which is necessary for its quantification, potentially compromising the accuracy of sulfur measurements. Additionally, the high reactivity of fluorine may lead to corrosion or damage of the instrument, further contributing to inaccuracies. The complex interactions between fluorine, sulfur, and other components of the silica shell could also interfere with the recovery and precise quantification of sulfur.

Therefore, to achieve reasonable control during polymerization and enhance grafting efficiency, the amount of ABDV used was selected based on previous studies[20, 21] and preliminary experiments. By adjusting the ratio of the comonomer BMA to ABDV, the polymerization conditions were optimized while keeping the concentrations of other components, such as the fluorescent indicator monomer, the template, and the RAFT-grafted particles (by weight), constant.

The optimization results are presented in Table S4. When the amount of ABDV was approx. 10% of BMA, the fluorescence change induced by the rebinding of FEX (0.7 mM) to the MIP was around 0.55. In contrast, reducing the amount of ABDV to 1% or 0.5% resulted in fluorescence changes of 0.35 and 0.01, respectively. The larger signal change at 10% ABDV indicates the formation of MIPs with higher specific recognition ability and was therefore used here for controlled MIP growth.

**Table S4.** Optimization of ABDV component in MIP synthesis

| MIP particles (experiment #) | BMA : ABDV | $I_{F,0}/I_{F,r}–I_F/I_{F,r}$ |
|---|---|---|
| **gMIP@rCS** (#1) | 1 : 0.10 | 0.55 |
| **gMIP@rCS** (#2) | 1 : 0.01 | 0.35 |
| **gMIP@rCS** (#3) | 1 : 0.005 | 0.01 |

## XIX. Optimization of Polymerisation Time

Based on our previous work,[20-22] we initially selected a polymerization time of 18 h at 50 °C, which has proven to yield well-defined polymer networks with similar MIP components. However, TEM characterization later revealed that the polymerization conditions resulted in a certain swelling of some of the polystyrene cores, which in turn led to the rupture of the silica shell and subsequent particle degradation. Moreover, the prolonged reaction time resulted in the formation of a substantial amount of junk polymer, which was not expected, as shown in Figure S9, below.

To resolve these issues, we conducted a few tests to determine the optimal reaction time for polymerization at 50 °C by monitoring the survival time of the core particles in the porogen (acetonitrile) at intervals of 3, 6, and 10 h. TEM images indicated that a polymerization time of 3 h avoided any swelling of the polystyrene core (Figure S10), maintaining the integrity of the core-shell structure. Additionally, the MIP particles produced under the optimized conditions exhibited improved specificity, with a discrimination factor of 6.5 (MIP against FEX to MIP against competitor AMPI), which is notably higher than the discrimination factor of 3.5 observed with the 18-h polymerization.

Furthermore, the MIP particles generated with a 3-h polymerization time were consistently reproducible (see Figure S16 below), and displayed well-defined morphology (Figure 6).

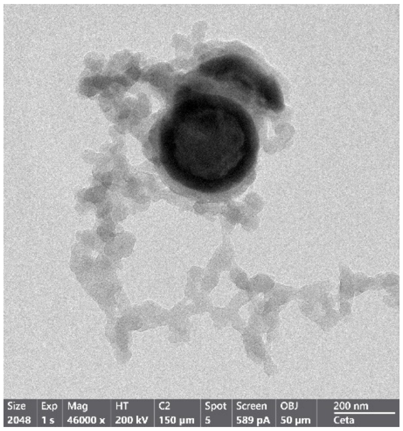


**Figure S9**. TEM image of **gMIP@rCS** synthesized via polymerization at 50 °C for 18 h.

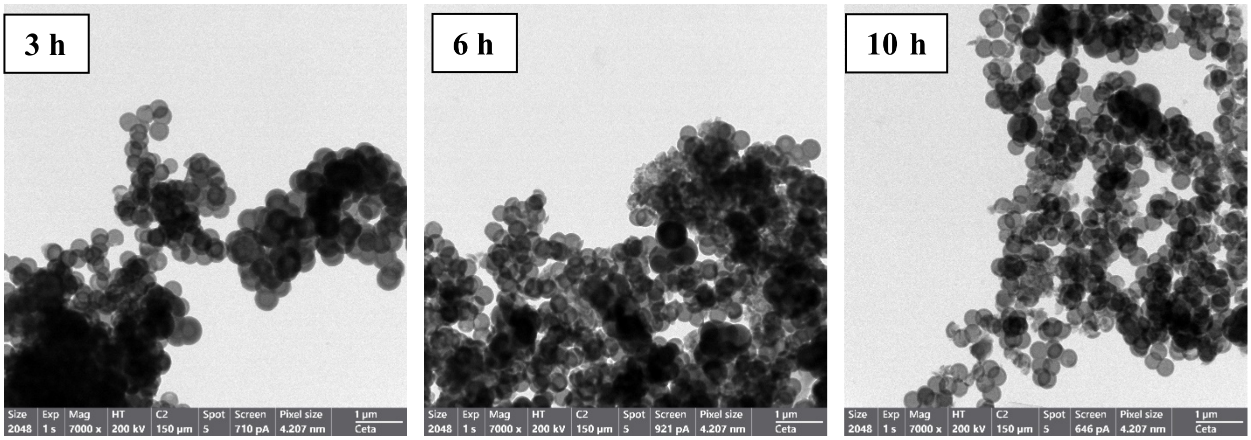


**Figure S10**. TEM images of **gMIP@rCS** synthesized at 50 °C with a polymerization time of 3 h (left), 6 h (middle) and 10 h (right).

## XX. Studies on the Origin of the Unusually Structured Emission of gMIP@rCS

To investigate the unusually structured emission of **gMIP@rCS**, excitation spectra were collected at different emission wavelengths. Figure S11 (left) reveals that the excitation spectrum observed at 575 nm was identical to that at 538 nm, indicating that the two emission bands originated from a single ground-state species, i.e., indicator monomer $\mathbf{M_1}$ that was embedded in the MIP shell.

Because also the absorption spectrum did not give a hint for a sizable formation of a species with an absorption maximum between those of $\mathbf{M_1}$ and **I**, see green spectrum in comparison to black and red spectrum in Figure 5, the formation of ground state dimers or even aggregates was considered less likely.

To verify this assumption, the concentration of $\mathbf{M_1}$ incorporated into the MIP shell was approximated from absorption measurements as well as the knowledge of the molar absorption coefficient, the MIP shell thickness and the composite particle's density, arriving at an average concentration of ca. 33 mM $\mathbf{M_1}$ in the MIP shell of **gMIP@rCS**. Because this concentration is roughly four orders of magnitude higher than that of $\mathbf{M_1}$ in the experiments in dilute solution (1 μM), the spectroscopic behavior of $\mathbf{M_1}$ was also investigated at such high concentrations in solution.

Absorption and emission spectra of the concentrated solution were recorded using microcuvettes with a pathlength of 10 μm, and a front-face geometry in the fluorometer (Figure S11, right). These experiments showed that despite a certain red-shift of the typical emission band of the dye at approx. 540 nm, which is due to the front-face spectrum being uncorrected, a new, intense shoulder appeared at ca. 580 nm (dashed lines, Figure S11, right), whereas the absorption spectra remained unaltered (solid lines, Figure S11, right), supporting our hypothesis of an absence of ground-state interactions between indicator molecules at such high concentrations.

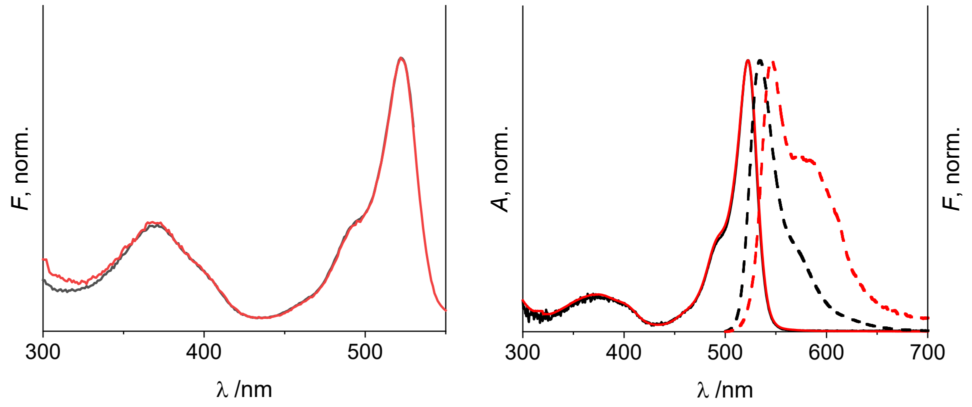


**Figure S11**. Left: excitation spectra of **gMIP@rCS** in acetonitrile observed at 538 nm (black) and 575 nm (red). Right: absorption (solid) and emission (dash) spectra of **M1** in MeCN at 1 µM (black) and 33 mM (red).

Having in mind the various possibilities for the formation of anomalous emission bands in BODIPY dyes,[23] we extracted the emission spectrum of the unknown species from the two spectra given in Figure S11 (right). Figure S12 details in brief how the differences due to instrumental settings that were necessary for the measurement of the dilute and the concentrated solution were taken into account.

Before starting the data treatment, the emission band obtained for dilute **M1** was converted to the energy scale and fitted to a progression of Gaussian bands, which nicely revealed that the neat emission of **M1** at dilute concentrations follows the typical polymethine pattern. The data treatment then led to a rather broad and unstructured band centered at ca. 590 nm for the unknown species, Figure S13.

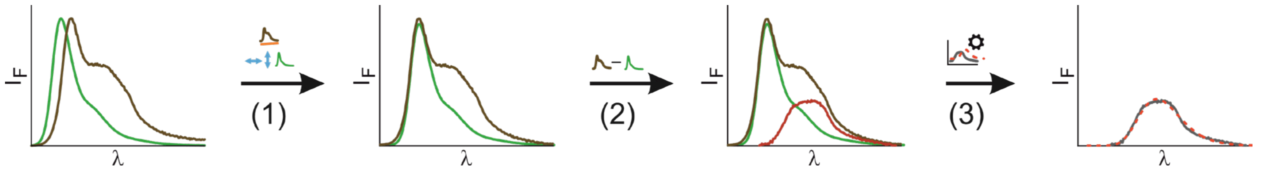


**Figure S12.** Data treatment to extract the unknown species from the spectra shown in Figure S11 (right). (1): baseline correction of brown spectrum and shifting of green spectrum in x and y direction to minimize the difference between the spectra in the blue region ≤540 nm. (2): Subtraction of green from brown spectrum to yield red spectrum. (3): Fit of red spectrum with a lognormal function to yield red dotted spectrum.

In agreement with the literature on BODIPY dimers, excimers and aggregates, see refs [23-25] and works cited therein, we tentatively attribute this band to excimers of **M1** that are closely enough positioned in the MIP shell and, because of their covalent fixation in the network, are limited with respect to diffusion.

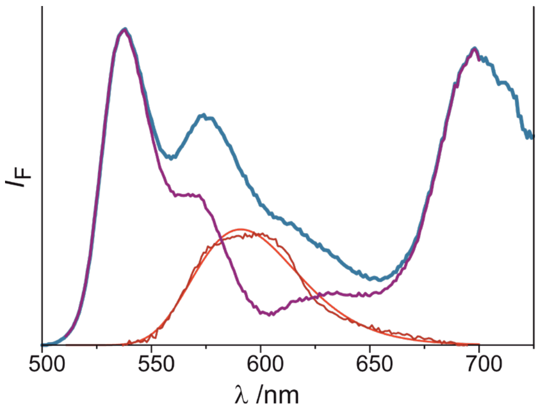


**Figure S13.** Reconstructed (brown) and fitted (red) spectrum of the unknown species and measured spectrum of **gMIP@rCS** (blue), plotted together with the difference spectrum (purple) of the blue and the red spectrum.

Concentration-dependent experiments of **M1** carried out in microcuvettes further revealed that at concentrations above ca. 10 mM, a second red-shifted band appears in emission whereas the absorption spectra remain unaltered, Figure S14.

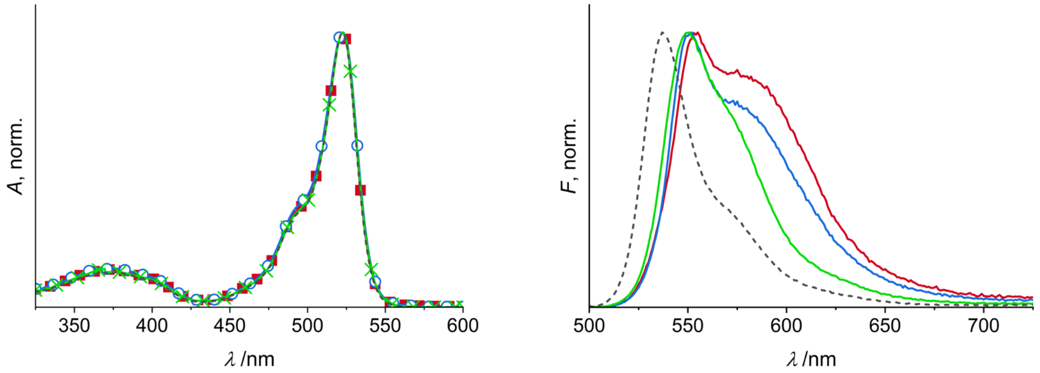


**Figure S14.** Normalized absorption (left) and fluorescence spectra (right) of **M1** in MeCN at 33 mM (red/squares), 25 mM (blue/circles), 13 mM (green/crosses) and 0.75 mM (black dotted); spectra recorded in cuvettes with a pathlength of 10 μm, in front-face geometry for fluorescence.

Because the microenvironment of the single **M1** units in the MIP shell in acetonitrile is presumably different to neat acetonitrile, leaving the polarity and refractive index that **M1** is located in unknown, the exact position, width and asymmetry of the excimer band in the polymer layer also remain unknown.

These factors result in a difference spectrum (purple, Figure S13) that does not show baseline-separated contributions of monomeric **M1** (black, Figure 5) and **I** (red, Figure 5). However, Figure S13 reveals that besides a certain minor contribution in the 600–650 nm region, the major species contributing to the emission of **gMIP@rCS** are monomers and excimers of **M1** and monomers of **I**, suggesting that a certain fraction of the covalently integrated **M1** indicators can interact in the excited state.

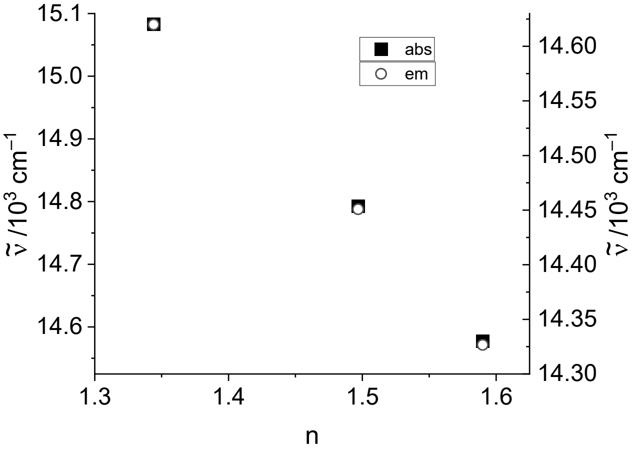


**Figure S15.** Plots of the absorption and emission maxima of **I** in MeCN, toluene, and polystyrene matrix vs refractive index of the media; refractive indices taken from ref [18] (MeCN, toluene) and refs [26, 27] (PS nanoparticles).

## XXI. Reproduction of gMIP@rCS

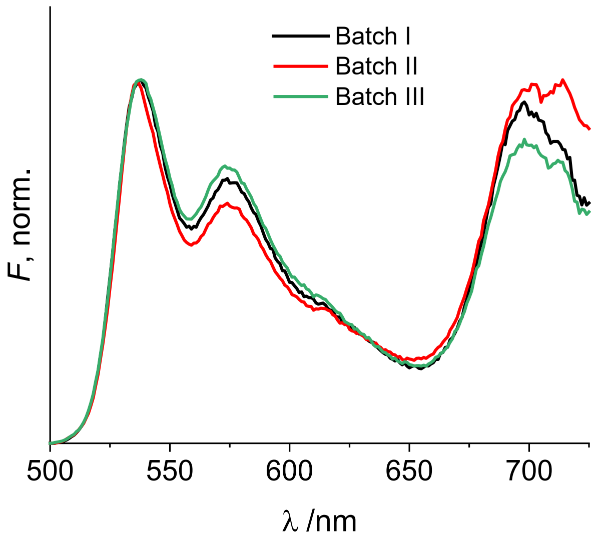


**Figure S16.** Emission spectra of three different batches of **gMIP@rCS** in MeCN, $\lambda_{exc}$: 375 nm.

## XXII. Fluorescence Changes of gMIP@rCS

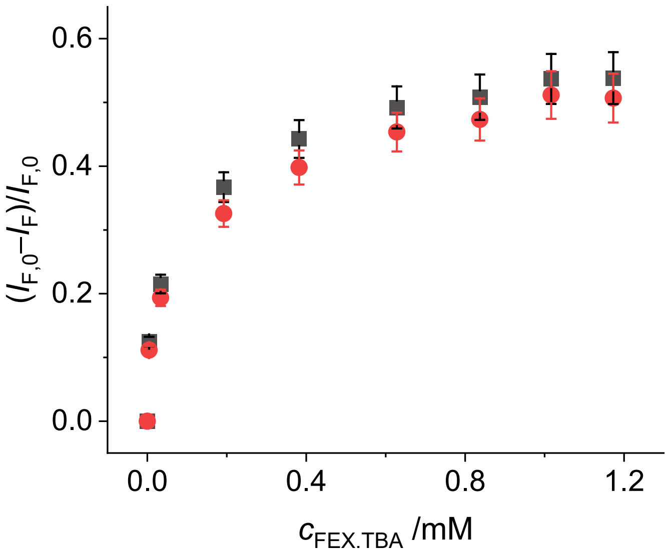


**Figure S17.** Fluorescence changes of **gMIP@rCS** towards FEX.TBA in acetonitrile at 538 nm (black squares) and 575 nm (red circles). Whereas $I_{F,0}$ is the maximum fluorescence emission in the absence of FEX.TBA, $I_F$ represents the maximum fluorescence emission upon each addition of FEX.TBA.

## XXIII. Determination of LOB and LOD / Demonstration of Reversibility

To calculate the limit of blank (LOB) and limit of detection (LOD) of **gMIP@rCS** versus FEX.TBA in acetonitrile, the blank was repeatedly measured three times, and incremental addition of FEX.TBA to the particle suspension, subsequently the data was collected and fitted with a Logistic function. The following formulas were used to determine the LOB and LOD.[28]

$LOB = \bar{x}_{blank} + 1.645 \cdot SD_{blank}$ , where $\bar{x}_{blank}$ is the mean concentration of the blank measurements (the concentration without analyte was fixed at 0.01 μM for fitting purposes) and $SD_{blank}$ is the standard deviation of the concentration of the blank measurements. The resulted LOB for **gMIP@rCS** is 71 nM.

$LOD = LOB + 1.645 \cdot SD_{lowest}$, where $SD_{lowest}$ is the standard deviation of the repeated measurements of the lowest concentration of analyte response. The resulted LOD for **gMIP@rCS** is 132 nM.

$LOQ = LOD + u_{mea}$, where $u_{mea}$ is the measurement uncertainty at the highest concentration of the analyte addition. The obtained LOQ for **gMIP@rCS** is 163 nM.

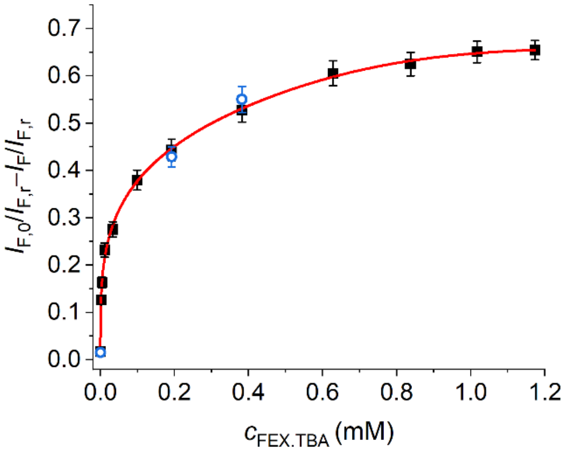


**Figure S18.** Logistic fitting of the changes in fluorescence intensity of **gMIP@rCS** at 538 nm relative to 690 nm ($I_{F,r}$) upon addition of FEX.TBA, where $I_{F,0}$ denotes no analyte addition and $I_F$ is the fluorescence intensity after each addition. Blue open circles are representative results of a reversibility experiment carried out in a cuvette: (i) 1.50 mL of a suspension of **gMIP@rCS** was pipetted into a cuvette and measured in the absence of analyte (0.0 mM); (ii) second, the measurement was repeated after 0.19 mL of a FEX.TBA stock solution has been added into the cuvette (0.4 mM); (iii) before the last measurement, 1.71 mL of the **gMIP@rCS** suspension was added into the cuvette (0.2 mM).

## XXIV. Fluorescence Responses of gMIP@rCS to Competitors

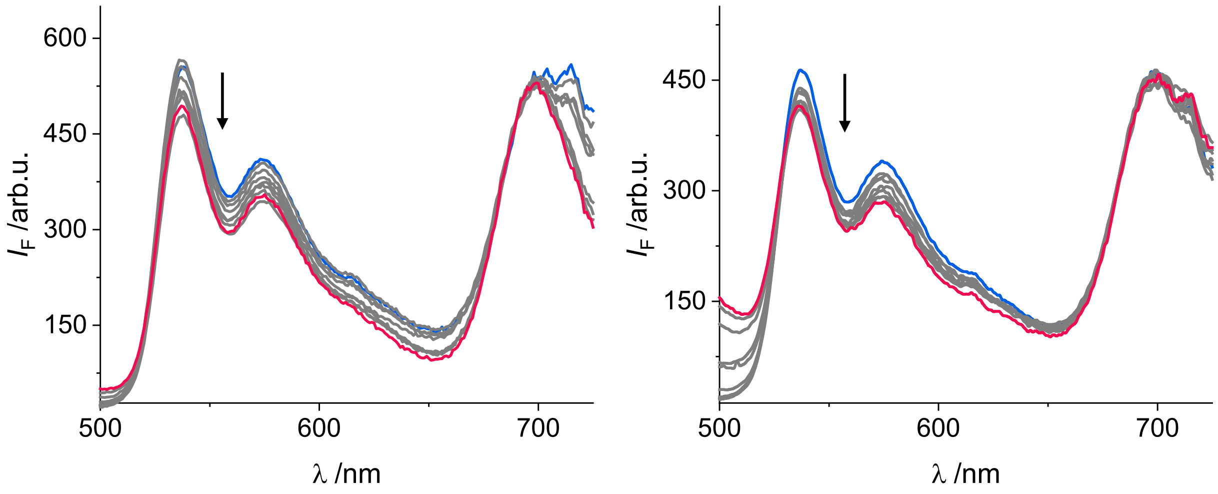


**Figure S19.** Changes in fluorescence intensity upon addition of AMPI.TBA (left, 0–1.2 mM) and AMOX.TBA (right, 0–1.2 mM) in MeCN (start and end point spectra shown in blue and red), $\lambda_{exc}$: 375 nm.

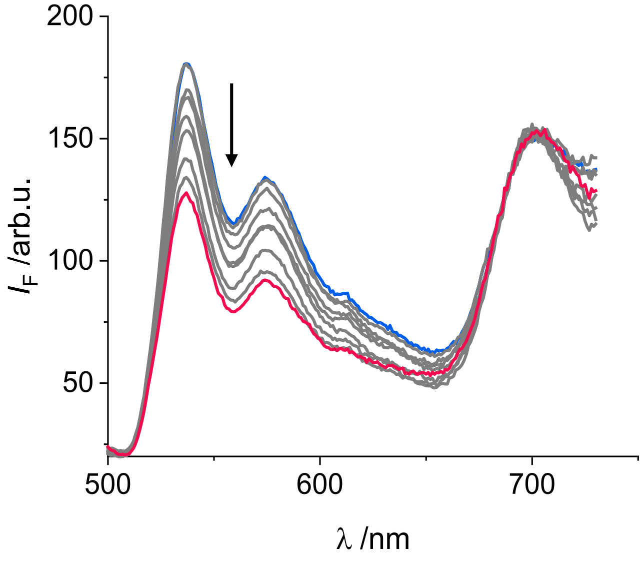


**Figure S20.** Changes in fluorescence intensity upon addition of NA.TBA (0–1.2 mM; start and end point spectra shown in blue and red) in MeCN; $\lambda_{exc}$: 450 nm, avoiding absorption of the analyte molecule at 375 nm.

## XXV. Fluorescence Responses of $M_1$ to Competitors

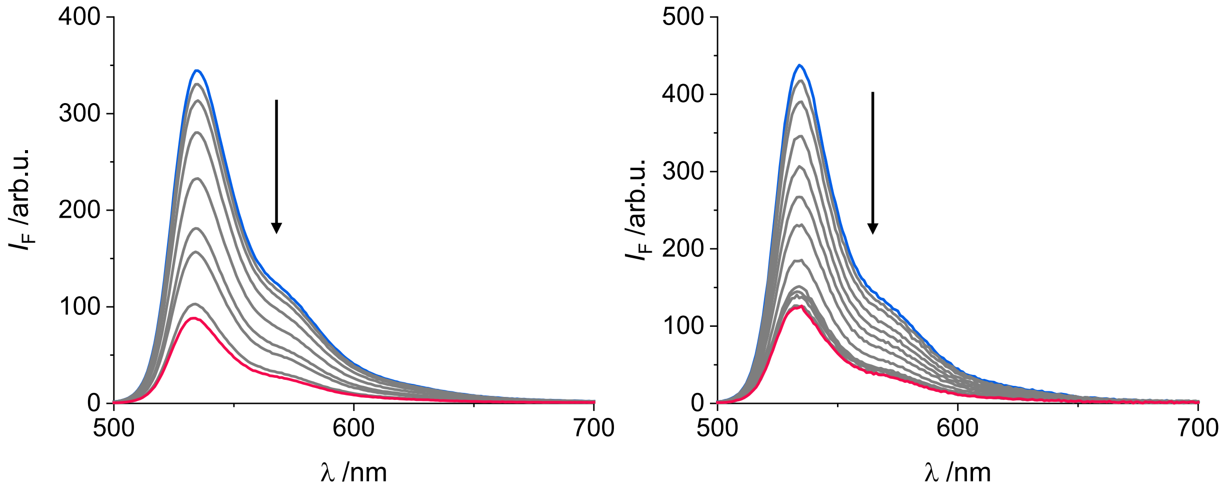


**Figure S21.** Fluorescence responses of $\mathbf{M_1}$ ($c$ = 1 μM) upon addition of AMPI.TBA (left, 0–0.8 mM) and AMOX.TBA (right, 0–0.97 mM) in MeCN (start and end point spectra shown in blue and red); $\lambda_{exc}$: 490 nm.

## XXVI. Comparison with Other Methods for the Detection of Fexofenadine

**Table S5.** Comparison of the reported methods for the determination of fexofenadine

| Method / detection | Materials / mechanism | Instrumentation | LOD (µM) | Range (µM) | Comments | Year | Ref. |
|---|---|---|---|---|---|---|---|
| HPLC / UV | Lisinopril as an internal standard / $C_{18}$ column as stationary phase / phosphate buffer containing 0.1% of 1-octane sulfonic acid sodium salt monohydrate with 1% triethylamine, pH 2.7 and as Mobile phase | HPLC, DAD | 0.04 | 0.2–100 | – | 2011 | [29] |
| HPLC / UV | Levocetirizine as an internal standard / $C_{18}$ column as stationary phase / acetonitrile and water as mobile phase | Spectrophotometer | 0.54 | 100–350 | – | 2018 | [30] |
| LC-MS / MS | Metoprolol as an internal standard / chirobiotic V column as stationary phase / methanol: ammonium acetate, pH 4.25 as mobile phase | HPLC, triple quad-MS with ESI | n.r. | 0.04–20 | – | 2020 | [31] |
| Potentiometry | Carbon paste electrodes doped with FEX molybdate / binding of FEX in membrane restricts access of ions to electrode | pH/mV meter / vessel | 0.01 | 5–10000 | Only high concentration range shown | 2022 | [32] |
| Potentiometry | Cyclodextrin (CD) ionophore in polymer membrane / binding of FEX in CD restricts access of lipophilic ions to electrode | pH/mV meter / vessel | 1.3 | 4.5–10000 | Interference of $Na^+$, $K^+$, $Ca^{2+}$ at concentrations higher than FEX | 2023 | [33] |
| Spectrophotometry | Bromophenol blue / color deepening by ion pair formation between FEX–$H^+$ and dye anion | Spectrophotometer / cuvette | 0.13 | 2.4–90 | Weak, non-specific binding | 2013 | [34] |
| Fluorescence | p-Chloranilic acid / charge transfer complexes between quinone and FEX | Fluorometer / cuvette | 0.28 | 0.40–7 | Weak, non-specific | 2012 | [35] |

| Method / detection | Materials / mechanism | Instrumentation | LOD (µM) | Range (µM) | Comments | Year | Ref. |
|---|---|---|---|---|---|---|---|
| Synchronous fluorescence | Sodium dodecyl sulphate / direct measurement of FEX–$H^+$ | Fluorometer / cuvette | 0.05 | 0.2–2.8 | UV excitation (295 and 286 nm) | 2022 | [36] |
| Fluorescence | Cilefa Pink B (fluorescein derivative) / quenching of dye fluorescence by ion pair formation between FEX–$H^+$ and dye anion | Fluorometer / cuvette | 0.05 | 0.2–4 | Weak, non-specific | 2022 | [37] |
| Fluorescence | Sulfuric acid / direct measurement of FEX–$H^+$ | Plate reader / well plate Fluorometer / cuvette | 0.02 | 0.02–1.00 | UV excitation (295 nm) | 2024 | [38] |
| Fluorescence | Fluorescent core-shell MIP / binding-induced fluorescence change | Fluorometer / cuvette | 0.13 | 0.16–1200 | see text | 2024 | This work |

## XXVII. Calculation of Measurement Uncertainties

Because of the multiplicative and quotient forms of the respective equations, and because correlations between the quantities are assumed to be negligible, summation of the squares of the relative uncertainties was performed.[39]

Preparation of 1 MIP suspension and 4 different analyte solutions:

Weighing of ca. 1 mg of MIP particles (balance Mettler Toledo 1 ± 0.01 mg); $u_{rel}^{w1}$ = 1 %

Dispersing in ca. 10 mL acetonitrile (Eppendorf Reference pipette purple 10 mL ± 0.06 mL); $u_{rel}^{d1}$ = 0.6 %

Weighing ca. 7 mg of analyte (balance Mettler Toledo ± 0.01 mg); $u_{rel}^{wa}$ = 0.14 %,

Dissolving in 2.7 mL acetonitrile (Eppendorf Reference pipette purple 5 mL ± 0.03 mL); $u_{rel}^{da}$ = 1.11%

Preparation of measurement solutions

Taking 750 µL of the MIP dispersion and diluting into 750 µL acetonitrile in 10 mm optical path length quartz cell (blue pipette 1000 µL ± 5.5 µL); $2* u_{rel}^{d2}$ = $2*$ 0.73 %; contribution from cell length (± 0.01 mm); $u_{rel}^{l}$ = 0.1 %

Addition of 1 µL analyte solution into the MIPs dispersion (Eppendorf Reference pipette 10 µL ± 0.025 µL); $2* u_{rel}^{d3}$ = $2*$ 2.50 %

Addition of 13 µL analyte solution into the MIPs dispersion (Eppendorf Reference pipette 100 µL ± 0.3 µL); $u_{rel}^{d4}$ = 2.31 %

Addition of 30 µL analyte solution into the MIPs dispersion (Eppendorf Reference pipette 100 µL ± 0.4 µL); $u_{rel}^{d5}$ = 1.33 %

Addition of 45 µL analyte solution into the MIPs dispersion (Eppendorf Reference pipette 100 µL ± 0.5 µL); $u_{rel}^{d6}$ = 1.11 %

Addition of 100 µL analyte solution into the MIPs dispersion (Eppendorf Reference pipette 100 µL ± 0.8 µL); $u_{rel}^{d7}$ = 0.80 %

Addition of 150 μL analyte solution into the MIPs dispersion (Eppendorf Reference pipette 1000 μL ± 3 μL); 4∗ $u_{rel}^{d8}$ = 4∗ 2 %

Repetition of measurements

$u_{rel}^{re}$ = 0.1 % − 3.8 %

Total relative uncertainties:

$$\sqrt{({u_{rel}^{w1}}^2 + {u_{rel}^{d1}}^2 + {u_{rel}^{wa}}^2 + {u_{rel}^{da}}^2 + 2*{u_{rel}^{d2}}^2 + {u_{rel}^{l}}^2 + 2*{u_{rel}^{d3}}^2 + {u_{rel}^{d4}}^2 + {u_{rel}^{d5}}^2 + {u_{rel}^{d6}}^2 + {u_{rel}^{d7}}^2 + 4*{u_{rel}^{d8}}^2 + {u_{rel}^{re}}^2)}$$

= 3.1 – 7.6 %

## XXVIII. $^1$H and $^{13}$C NMR Spectra of BODIPYs

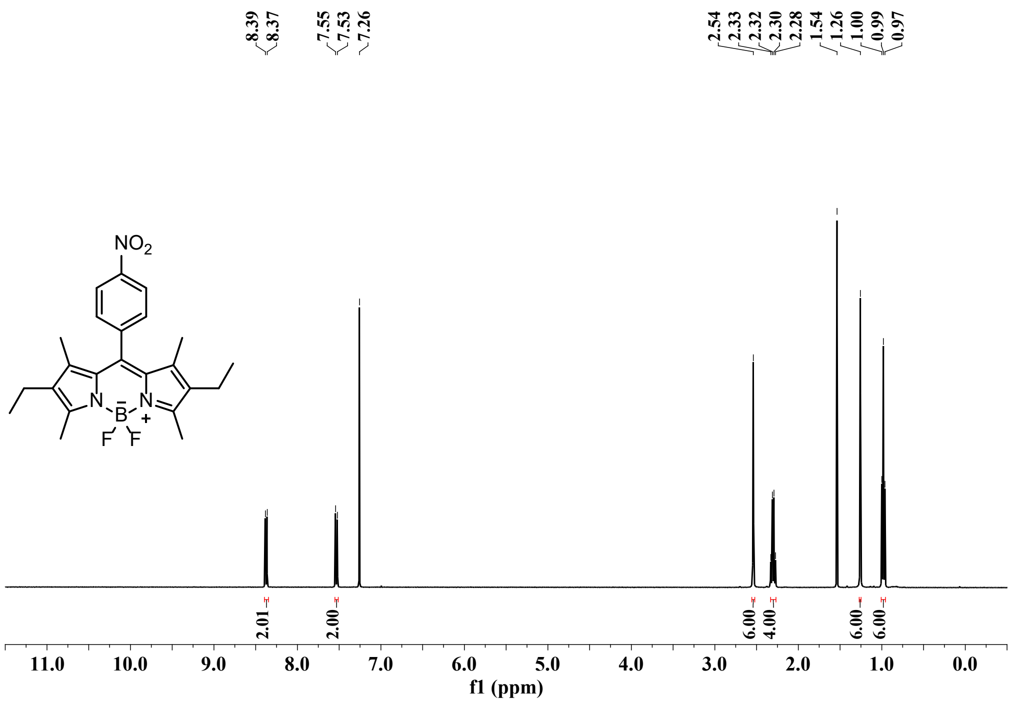


**Figure S22.** $^1$H NMR spectrum of *meso*-(4-nitrophenyl)-BODIPY **3** in $CDCl_3$.

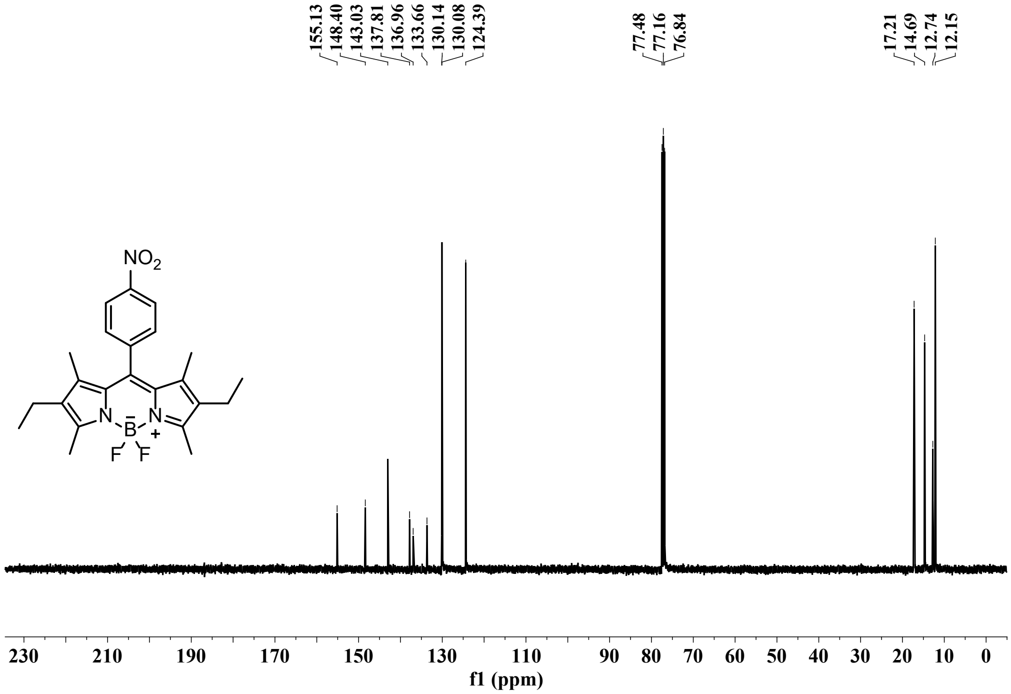


**Figure S23.** $^{13}$C NMR spectrum of *meso*-(4-nitrophenyl)-BODIPY **3** in $CDCl_3$.

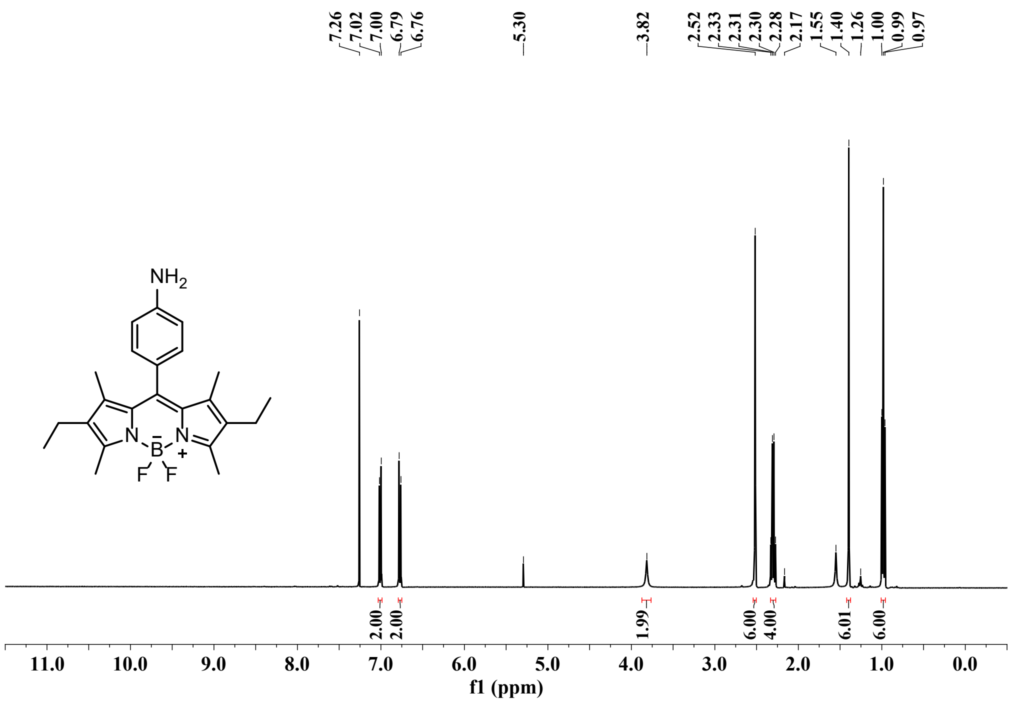


**Figure S24.** $^{1}$H NMR spectrum of *meso*-(4-aminophenyl)-BODIPY **2** in $CDCl_3$.

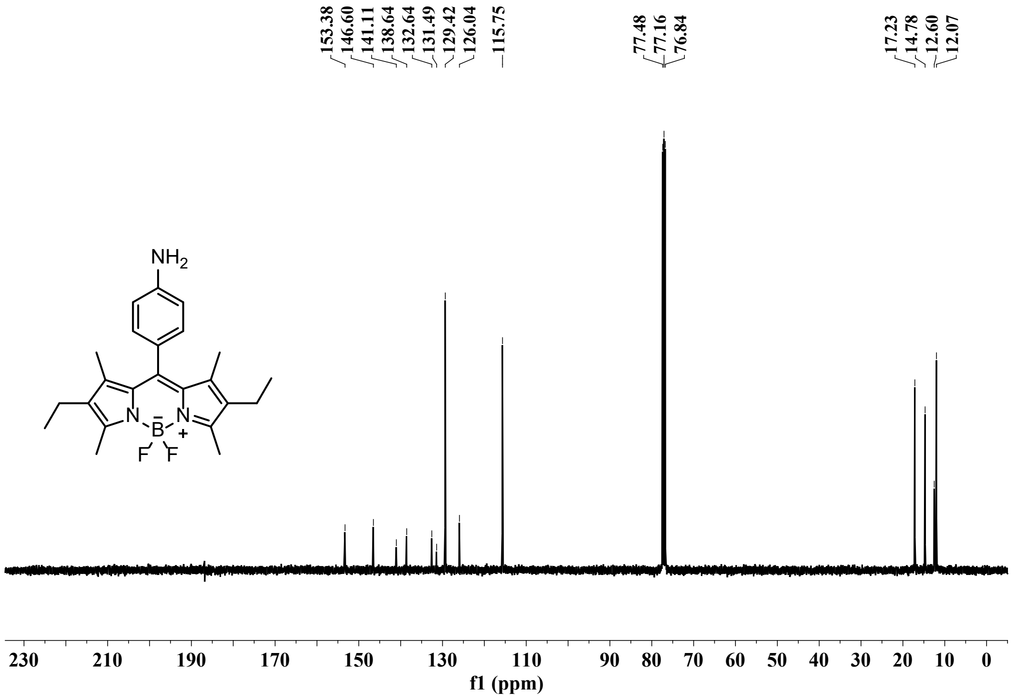


**Figure S25.** $^{13}$C NMR spectrum of *meso*-(4-aminophenyl)-BODIPY **2** in $CDCl_3$.

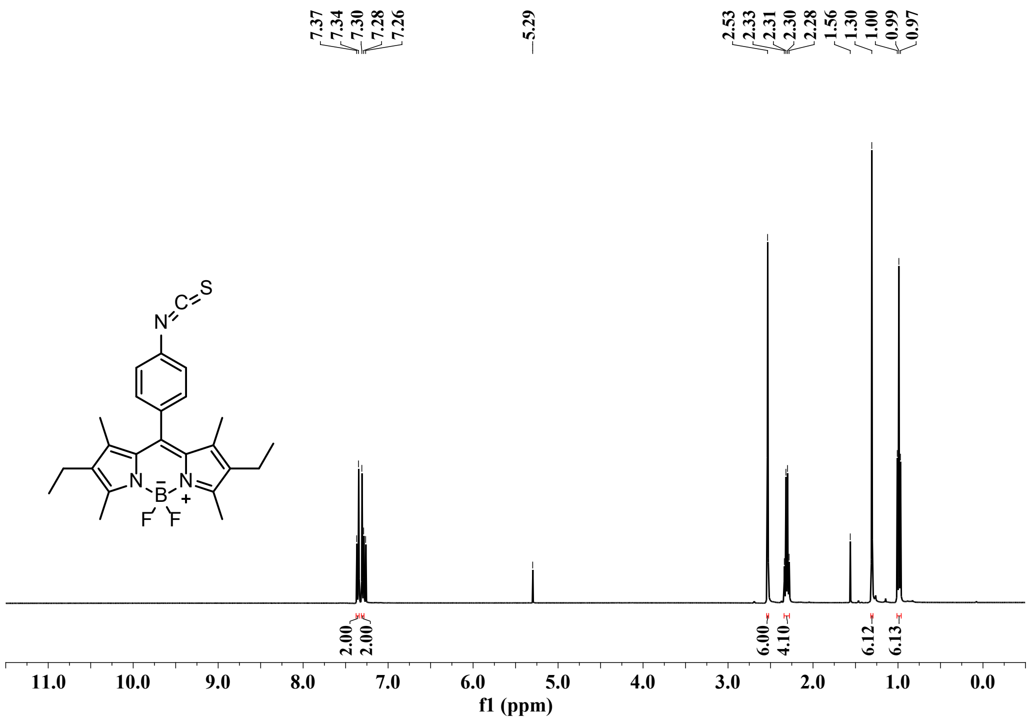


**Figure S26.** $^{1}H$ NMR spectrum of *meso*-(4-isothiocyanatophenyl)-BODIPY **1** in $CDCl_3$**.**

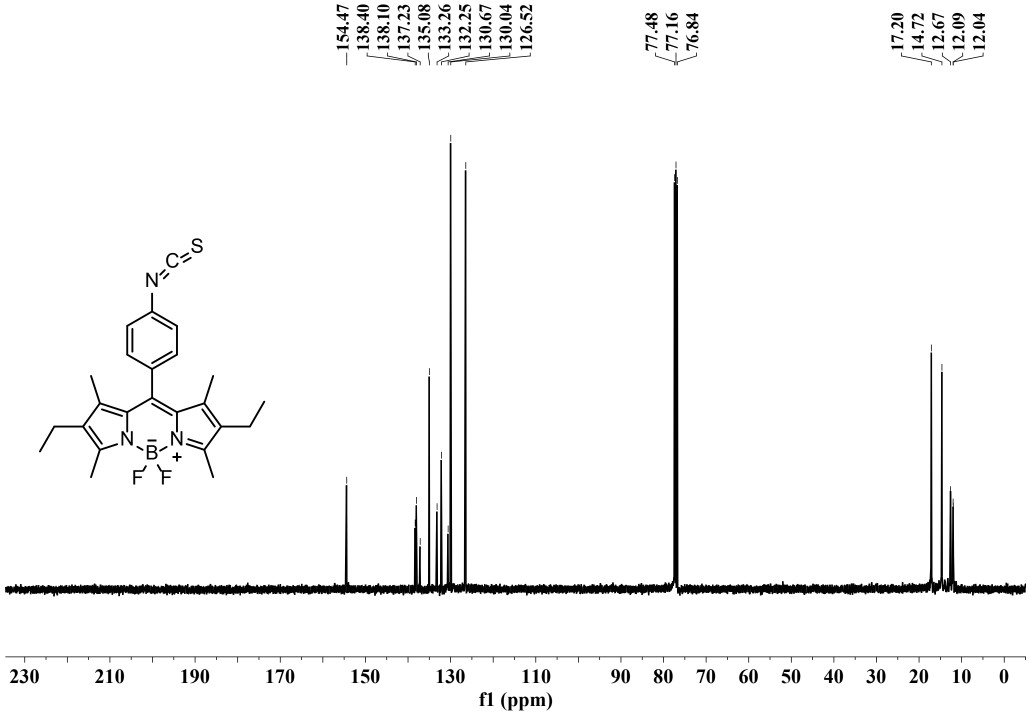


**Figure S27.** $^{13}C$ NMR spectrum of *meso*-(4-isothiocyanatophenyl)-BODIPY **1** in $CDCl_3$.

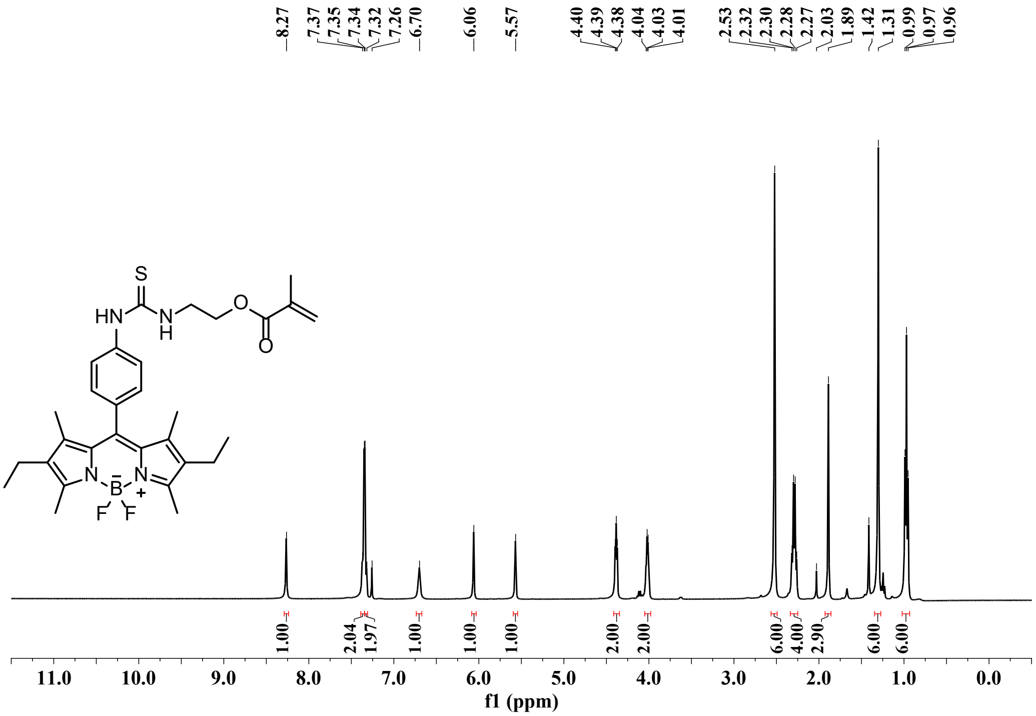


**Figure S28.** $^{1}H$ NMR spectrum of indicator monomer **M₁** in $CDCl_3$.

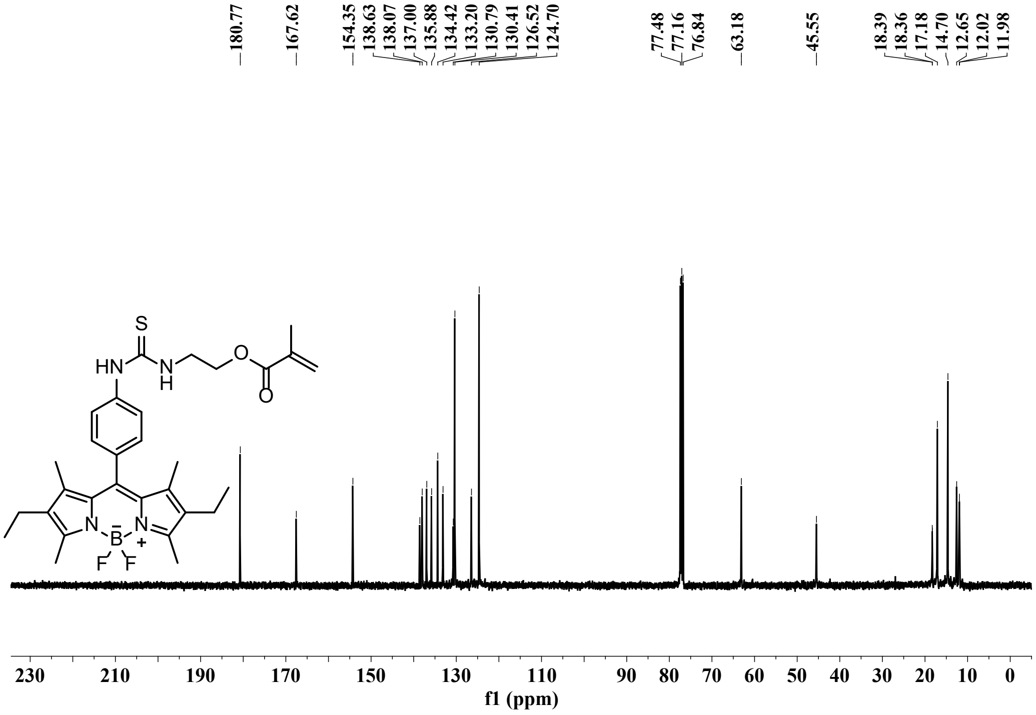


**Figure S29.** $^{13}C$ NMR spectrum of indicator monomer **M₁** in $CDCl_3$.

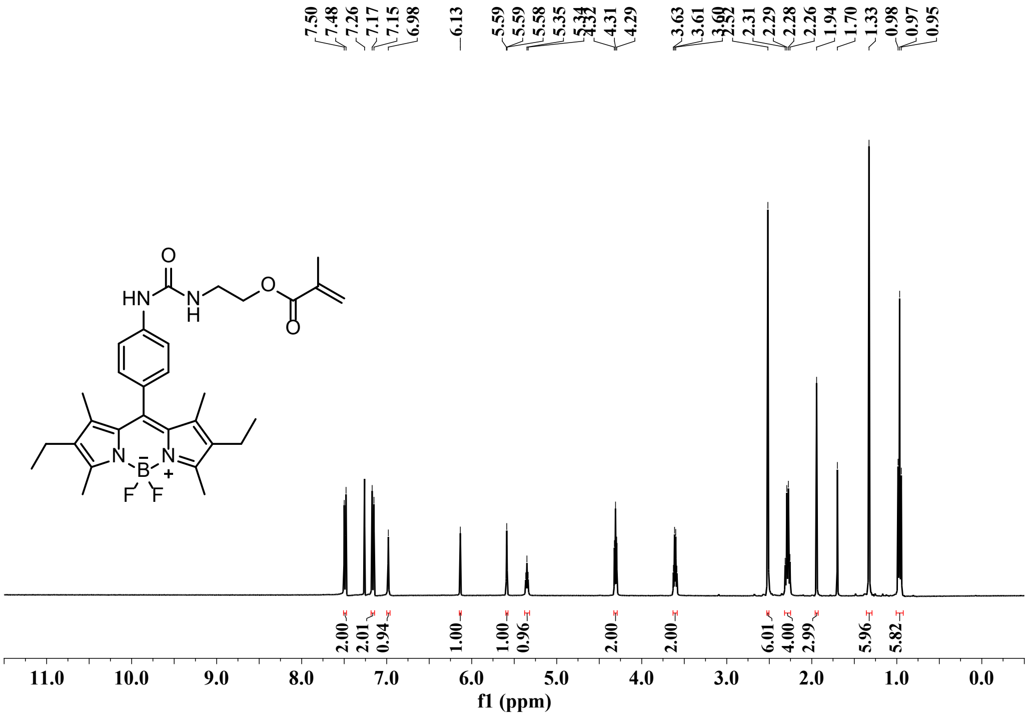


**Figure S30.** $^{1}H$ NMR spectrum of indicator monomer **M₂** in $CDCl_3$.

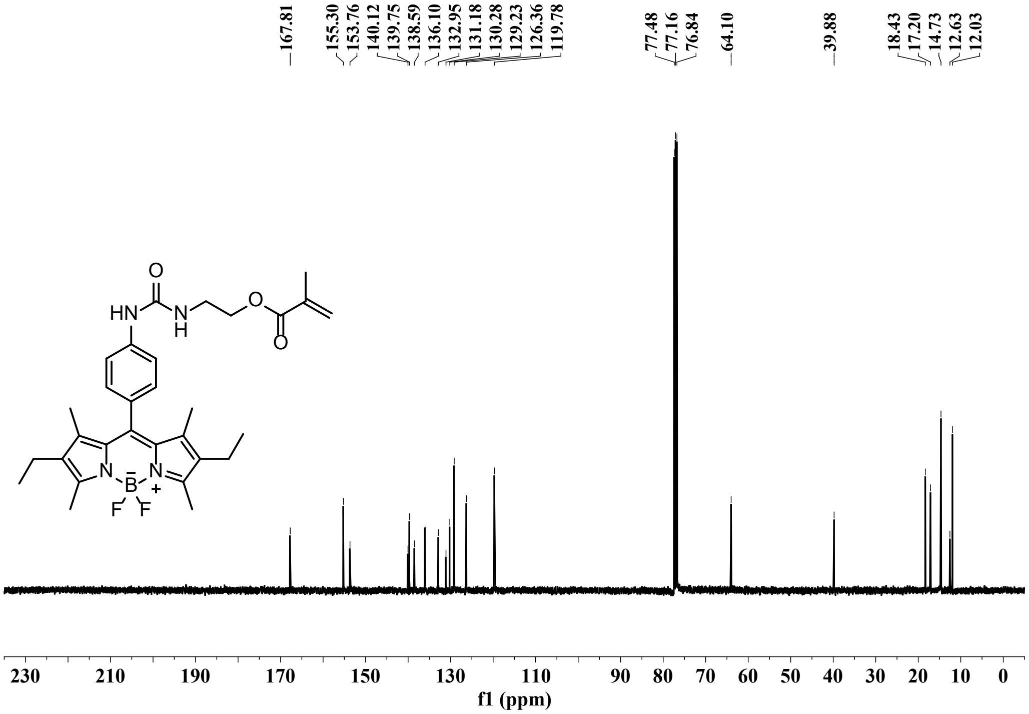


**Figure S31.** $^{13}C$ NMR spectrum of indicator monomer **M₂** in $CDCl_3$.

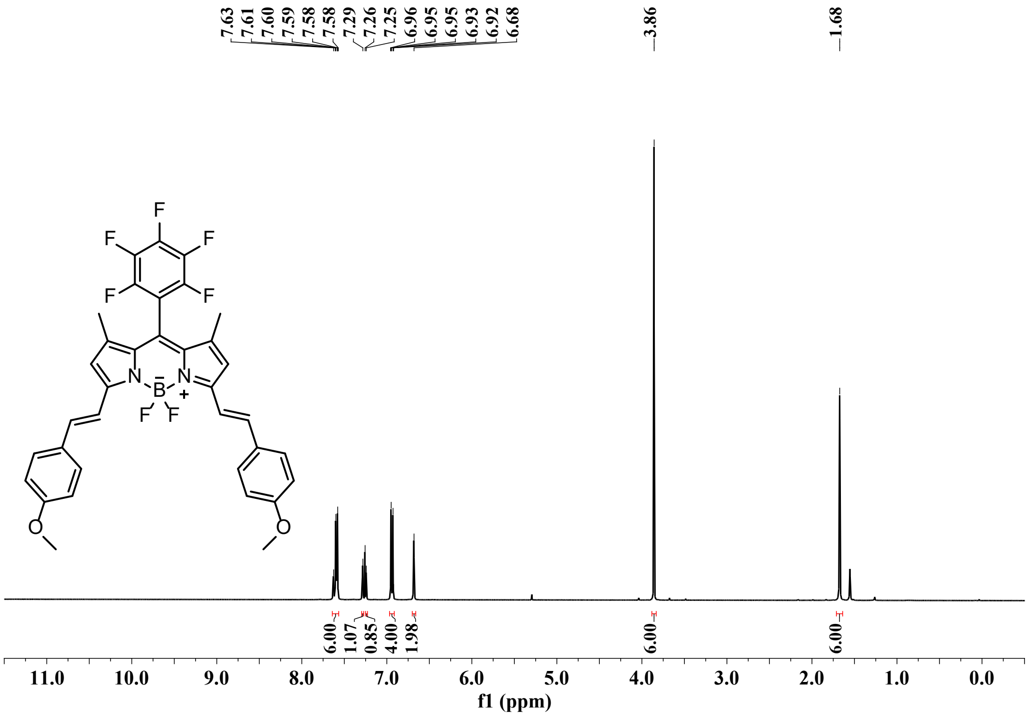


**Figure S32.** $^{1}H$ NMR spectrum of red BODIPY **I** in $CDCl_3$.

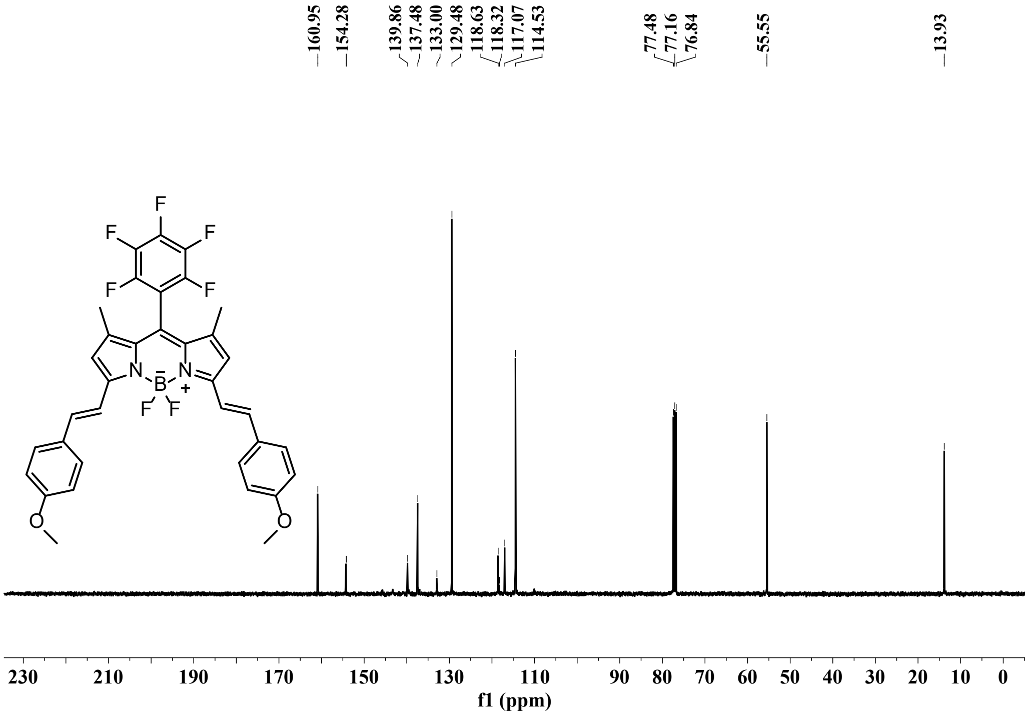


**Figure S33.** $^{13}C$ NMR spectrum of ***red BODIPY I*** in $CDCl_3$.

## XXIX. Detailed Results of Quantum Chemical Calculations Following Various Refs[8-13]

Results of calculations using the B3LYP functional with the 6-31G basis set for geometry optimization and the CAM-B3YLP functional for vertical excitations (in gas phase).

| **Species**[a] | **Basis set** | $\mu_g$ /D | $\lambda_{S1}$ /nm (*f*) | $\Delta\mu_{e-g}$ /D | $\lambda_{S2}$ /nm (*f*) | $\Delta\mu_{e-g}$ /D | $\lambda_{S3}$ /nm (*f*) | $\Delta\mu_{e-g}$ /D | $\lambda_{S4}$ /nm (*f*) | $\Delta\mu_{e-g}$ /D |
|---|---|---|---|---|---|---|---|---|---|---|
| **M$_1$** | 6-31G | 8.9 | 425.2 (0.576) | −1.6 | 334.5 (0.120) | −1.0 | 316.1 (0.002) | −0.3 | 313.6 (0.071) | −0.1 |
| **M$_1$**A/T | 6-31G | 15.0 | 422.8 (0.571) | −0.1 | 353.7 (0.024) | **+15.2** | 332.9 (0.111) | −4.1 | 312.0 (0.001) | **+22.6** |
| **M$_1$** | 6-31G(d) | 8.3 | 435.9 (0.559) | −1.5 | 340.8 (0.103) | −1.0 | 319.8 (0.002) | −0.2 | 318.4 (0.059) | −0.2 |
| **M$_1$**A/T | 6-31G(d) | 14.7 | 433.4 (0.553) | +0.1 | 355.1 (0.024) | **+14.6** | 339.1 (0.095) | −4.0 | 314.2 (0.040) | +4.4 |
| **M$_1$** | 6-311G(d,p) | 8.5 | 440.0 (0.569) | −1.4 | 341.8 (0.094) | −0.9 | 322.6 (0.002) | −0.2 | 318.2 (0.061) | −0.1 |
| **M$_1$**A/T | 6-311G(d,p) | 14.6 | 437.7 (0.562) | +0.1 | 353.7 (0.025) | **+14.6** | 340.2 (0.086) | −3.9 | 314.1 (0.050) | +4.1 |

[a] Color code: = BODIPY-centered, = *meso* group-centered, = occupied MO on BODIPY, unoccupied MO on *meso* group, = unoccupied MO on *meso* group, occupied MO on BODIPY, blue = trend agrees with experiment, red = trend disagrees with experiment, **bold** = large dipole moment change indicative of charge-transfer transition.

Results of calculations using the B3LYP functional with the 6-31+G(d) basis set for geometry optimization and the CAM-B3YLP functional for vertical excitations (in gas phase).

| **Species**[a] | **Basis set** | $\mu_g$ /D | $\lambda_{S1}$ /nm (*f*) | $\Delta\mu_{e-g}$ /D | $\lambda_{S2}$ /nm (*f*) | $\Delta\mu_{e-g}$ /D | $\lambda_{S3}$ /nm (*f*) | $\Delta\mu_{e-g}$ /D | $\lambda_{S4}$ /nm (*f*) | $\Delta\mu_{e-g}$ /D |
|---|---|---|---|---|---|---|---|---|---|---|
| **M$_1$** | 6-31+G(d) | 8.0 | 435.0 (0.581) | −1.5 | 335.5 (0.079) | −0.9 | 319.3 (0.002) | −0.3 | 313.1 (0.066) | 0.0 |
| **M$_1$**A/T | 6-31+G(d) | 15.3 | 432.6 (0.559) | −0.2 | 354.4 (0.083) | **+13.7** | 333.5 (0.071) | −2.4 | 310.5 (0.012)[c] | +5.2 |
| **M$_1$** | 6-311++G(d,p) | 7.9 | 436.7 (0.585) | −1.5 | 336.0 (0.075) | −0.8 | 321.4 (0.001) | −0.3 | 313.6 (0.064) | 0.0 |
| **M$_1$**A/T | 6-311++G(d,p) | 15.2 | 434.4 (0.563) | −0.2 | 353.5 (0.083) | **+13.7** | 334.1 (0.068) | −2.4 | 311.8 (0.001)[c] | +5.8 |

[a] Color code: = BODIPY-centered, = *meso* group-centered, = occupied MO on BODIPY, unoccupied MO on *meso* group, = unoccupied MO on *meso* group, occupied MO on BODIPY, blue = trend agrees with experiment, red = trend disagrees with experiment, **bold** = large dipole moment change indicative of charge-transfer transition. [c] Split (60:40) transition involving a *meso* group-centered transition and a transition from the *meso* group to BODIPY.

Results of calculations using the B3LYP functional with the 6-311G(d,p) basis set for geometry optimization and the CAM-B3YLP functional for vertical excitations (in MeCN via IECPM).

| **Species[a]** | **Basis set** | $\mu_g$ **/D** | $\lambda_{S1}$ **/nm (*f*)** | $\Delta\mu_{e-g}$ **/D** | $\lambda_{S2}$ **/nm (*f*)** | $\Delta\mu_{e-g}$ **/D** | $\lambda_{S3}$ **/nm (*f*)** | $\Delta\mu_{e-g}$ **/D** | $\lambda_{S4}$ **/nm (*f*)** | $\Delta\mu_{e-g}$ **/D** |
|---|---|---|---|---|---|---|---|---|---|---|
| **$M_1$** | 6-311++G(d,p) | 11.7 | 489.6 (0.884) | −1.3 | 339.3 (0.086) | −0.3 | 315.2 (0.166) | +1.3 | 311.3 (0.000) | **+16.2** |
| **$M_1$**A/T | 6-311++G(d,p) | 26.9 | 487.9 (0.881) | −0.4 | 346.7 (0.014) | +**11.0** | 338.4 (0.085) | −0.1 | 313.6 (0.151) | −0.1 |

[a] Color code: □ = BODIPY-centered, □ = *meso* group-centered, ◫ = occupied MO on BODIPY, unoccupied MO on *meso* group, ◫ = unoccupied MO on *meso* group, occupied MO on BODIPY, blue = trend agrees with experiment, red = trend disagrees with experiment, **bold** = large dipole moment change indicative of charge-transfer transition

Results of calculations using the B3LYP functional with the 6-311G(d,p) basis set for geometry optimization and the M06-2X functional for vertical excitations (in MeCN via IECPM).

| **Species[a]** | **Basis set** | $\mu_g$ **/D** | $\lambda_{S1}$ **/nm (*f*)** | $\Delta\mu_{e-g}$ **/D** | $\lambda_{S2}$ **/nm (*f*)** | $\Delta\mu_{e-g}$ **/D** | $\lambda_{S3}$ **/nm (*f*)** | $\Delta\mu_{e-g}$ **/D** | $\lambda_{S4}$ **/nm (*f*)** | $\Delta\mu_{e-g}$ **/D** |
|---|---|---|---|---|---|---|---|---|---|---|
| **$M_1$** | 6-311++G(d,p) | 11.5 | 491.7 (0.872) | −1.4 | 339.2 (0.096) | −0.1 | 320.7 (0.039) | **+17.9** | 315.0 (0.126) | +0.5 |
| **$M_1$**A/T | 6-311++G(d,p) | 26.8 | 490.2 (0.870) | −0.4 | 356.9 (0.011) | **+11.3** | 338.4 (0.154) | 0.0 | 314.2 (0.154) | −0.1 |

[a] Color code: □ = BODIPY-centered, □ = *meso* group-centered, ◫ = occupied MO on BODIPY, unoccupied MO on *meso* group, ◫ = unoccupied MO on *meso* group, occupied MO on BODIPY, □ = mixed fragments involved, blue = trend agrees with experiment, red = trend disagrees with experiment, **bold** = large dipole moment change indicative of charge-transfer transition

Results of calculations using the M06-2X functional with the 6-31+G(d) basis set for geometry optimization and the M06-2X functional for vertical excitations (in gas phase).

| **Species[a]** | **Basis set** | $\mu_g$ **/D** | $\lambda_{S1}$ **/nm (*f*)** | $\Delta\mu_{e-g}$ **/D** | $\lambda_{S2}$ **/nm (*f*)** | $\Delta\mu_{e-g}$ **/D** | $\lambda_{S3}$ **/nm (*f*)** | $\Delta\mu_{e-g}$ **/D** | $\lambda_{S4}$ **/nm (*f*)** | $\Delta\mu_{e-g}$ **/D** |
|---|---|---|---|---|---|---|---|---|---|---|
| **$M_1$** | 6-31+G(d) | 7.9 | 432.7 (0.564) | −1.7 | 329.1 (0.085) | −1.0 | 321.6 (0.004) | 0.0 | 308.2 (0.086) | +1.0 |
| **$M_1$**A/T | 6-31+G(d) | 12.3 | 431.5 (0.555) | +0.4 | 354.6 (0.137) | **+14.1** | 327.0 (0.079) | −3.9 | 319.7 (0.005) | **+11.2** |

[a] Color code: □ = BODIPY-centered, □ = *meso* group-centered, ◫ = occupied MO on BODIPY, unoccupied MO on *meso* group, ◫ = unoccupied MO on *meso* group, occupied MO on BODIPY, □ = mixed fragments involved, blue = trend agrees with experiment, red = trend disagrees with experiment, **bold** = large dipole moment change indicative of charge-transfer transition

Results of calculations using the M06-2X functional with the 6-31G(d) basis set for geometry optimization and the M06-2X functional for vertical excitations (in MeCN via IECPM).

| **Species**[a] | **Basis set** | $\mu_g$ **/D** | $\lambda_{S1}$ **/nm (*f*)** | $\Delta\mu_{e-g}$ **/D** | $\lambda_{S2}$ **/nm (*f*)** | $\Delta\mu_{e-g}$ **/D** | $\lambda_{S3}$ **/nm (*f*)** | $\Delta\mu_{e-g}$ **/D** | $\lambda_{S4}$ **/nm (*f*)** | $\Delta\mu_{e-g}$ **/D** |
|---|---|---|---|---|---|---|---|---|---|---|
| **M$_1$** | 6-311+G(2d,p) | 11.3 | 489.3 (0.864) | −1.6 | 335.9 (0.096) | −0.5 | 321.4 (0.203) | **+16.5** | 312.5 (0.085) | +0.3 |
| **M$_1$**A/T | 6-311+G(2d,p) | 19.6 | 488.2 (0.855) | −0.1 | 356.8 (0.303) | **+9.9** | 335.0 (0.095) | −1.2 | 312.1 (0.127) | +1.1 |

[a] Color code: = BODIPY-centered, = *meso* group-centered, = occupied MO on BODIPY, unoccupied MO on *meso* group, = unoccupied MO on *meso* group, occupied MO on BODIPY, = mixed fragments involved, blue = trend agrees with experiment, red = trend disagrees with experiment, **bold** = large dipole moment change indicative of charge-transfer transition

# XXX. References


(1) Xu, J.; Zhu, L.; Wang, Q.; Zeng, L.; Hu, X.; Fu, B.; Sun, Z. meso-C6F5 substituted BODIPYs with distinctive spectroscopic properties and their application for bioimaging in living cells. *Tetrahedron* **2014**, *70* (35), 5800-5805. DOI: 10.1016/j.tet.2014.06.040.

(2) Nandiyanto, A. B. D.; Suhendi, A.; Ogi, T.; Iwaki, T.; Okuyama, K. Synthesis of additive-free cationic polystyrene particles with controllable size for hollow template applications. *Colloids Surf., A* **2012**, *396*, 96-105. DOI: 10.1016/j.colsurfa.2011.12.048.

(3) Descalzo, A. B.; Xu, H.-J.; Xue, Z.-L.; Hoffmann, K.; Shen, Z.; Weller, M. G.; You, X.-Z.; Rurack, K. Phenanthrene-fused boron−dipyrromethenes as bright long-wavelength fluorophores. *Org. Lett.* **2008**, *10* (8), 1581-1584. DOI: 10.1021/ol800271e.

(4) Tobias, C.; Climent, E.; Gawlitza, K.; Rurack, K. Polystyrene microparticles with convergently grown mesoporous silica shells as a promising tool for multiplexed bioanalytical assays. *ACS Appl. Mater. Interfaces* **2021**, *13* (1), 207-218. DOI: 10.1021/acsami.0c17940.

(5) Sarma, D.; Gawlitza, K.; Rurack, K. Polystyrene core–Silica shell particles with defined nanoarchitectures as a versatile platform for suspension array technology. *Langmuir* **2016**, *32* (15), 3717-3727. DOI: 10.1021/acs.langmuir.6b00373.

(6) Würth, C.; Grabolle, M.; Pauli, J.; Spieles, M.; Resch-Genger, U. Relative and absolute determination of fluorescence quantum yields of transparent samples. *Nat. Protoc.* **2013**, *8* (8), 1535-1550. DOI: 10.1038/nprot.2013.087.

(7) Rurack, K.; Spieles, M. Fluorescence Quantum Yields of a Series of Red and Near-Infrared Dyes Emitting at 600−1000 nm. *Anal. Chem.* **2011**, *83* (4), 1232-1242. DOI: 10.1021/ac101329h.

(8) Nakano, K.; Konishi, T.; Imamura, Y. Estimation of maximum absorption wavelength of polymethine dyes in visible and near-infrared region based on time-dependent density functional theory. *Chem. Phys.* **2019**, *518*, 15-24. DOI: 10.1016/j.chemphys.2018.11.002.

(9) Fabian, J. TDDFT-calculations of Vis/NIR absorbing compounds. *Dyes Pigm.* **2010**, *84* (1), 36-53. DOI: 10.1016/j.dyepig.2009.06.008.

(10) Matulis, V. E.; Ragoyja, E. G.; Ivashkevich, O. A. Accurate theoretical prediction of optical properties of BODIPY dyes. *Int. J. Quantum Chem.* **2020**, *120* (9), e26159. DOI: 10.1002/qua.26159.

(11) Le Guennic, B.; Jacquemin, D. Taking Up the Cyanine Challenge with Quantum Tools. *Acc. Chem. Res.* **2015**, *48* (3), 530-537. DOI: 10.1021/ar500447q.

(12) Kachkovsky, O. D.; Naumenko, A. P.; Borisyuk, V. I.; Obernikhina, N. V.; Slominskiy, Y. L. Nature of Lowest Electron Transitions in Anionic Polymethine Dyes with Keto-Containing Terminal Groups. *Nanosistemi Nanomater. Nanotehnologii* **2022**, *20* (2), 473-486. DOI: 10.15407/nnn.20.02.473.

(13) Chibani, S.; Laurent, A. D.; Le Guennic, B.; Jacquemin, D. Improving the Accuracy of Excited-State Simulations of BODIPY and Aza-BODIPY Dyes with a Joint SOS-CIS(D) and TD-DFT Approach. *J. Chem. Theory Comput.* **2014**, *10* (10), 4574-4582. DOI: 10.1021/ct500655k.

(14) Wagner, S.; Zapata, C.; Wan, W.; Gawlitza, K.; Weber, M.; Rurack, K. Role of Counterions in Molecularly Imprinted Polymers for Anionic Species. *Langmuir* **2018**, *34* (23), 6963-6975. DOI: 10.1021/acs.langmuir.8b00500.

(15) Kollmannsberger, M.; Rurack, K.; Resch-Genger, U.; Daub, J. Ultrafast charge transfer in amino-substituted boron dipyrromethene dyes and its inhibition by cation complexation: A new design concept for highly sensitive fluorescent probes. *J. Phys. Chem. A* **1998**, *102* (50), 10211-10220. DOI: 10.1021/jp982701c.

(16) Karelson, M. M.; Zerner, M. C. Theoretical treatment of solvent effects on electronic spectroscopy. *J. Phys. Chem.* **1992**, *96* (17), 6949-6957. DOI: 10.1021/j100196a019.

(17) Maus, M.; Rettig, W.; Bonafoux, D.; Lapouyade, R. Photoinduced Intramolecular Charge Transfer in a Series of Differently Twisted Donor−Acceptor Biphenyls As Revealed by Fluorescence. *J. Phys. Chem. A* **1999**, *103* (18), 3388-3401. DOI: 10.1021/jp9905023.

(18) Reichardt, C.; Welton, T. *Solvents and Solvent Effects in Organic Chemistry*; Wiley-VCH, 2011. DOI: 10.1002/9783527632220.

(19) Thordarson, P. Determining association constants from titration experiments in supramolecular chemistry. *Chem. Soc. Rev.* **2011**, *40* (3), 1305-1323. DOI: 10.1039/C0CS00062K.

(20) Wan, W.; Descalzo, A. B.; Shinde, S.; Weißhoff, H.; Orellana, G.; Sellergren, B.; Rurack, K. Ratiometric Fluorescence Detection of Phosphorylated Amino Acids Through Excited-State Proton Transfer by Using Molecularly Imprinted Polymer (MIP) Recognition Nanolayers. *Chem. Eur. J.* **2017**, *23* (63), 15974-15983. DOI: 10.1002/chem.201703041.

(21) Sun, Y.; Gawlitza, K.; Valderrey, V.; Bell, J.; Rurack, K. Polymerizable BODIPY probe crosslinker for the molecularly imprinted polymer-based detection of organic carboxylates via fluorescence. *Mater. Adv.* **2024**, *5* (9), 3783-3793. DOI: 10.1039/d3ma00476g.

(22) Wan, W.; Biyikal, M.; Wagner, R.; Sellergren, B.; Rurack, K. Fluorescent sensory microparticles that "light-up" consisting of a silica core and a molecularly imprinted polymer (MIP) shell. *Angew. Chem. Int. Ed.* **2013**, *52* (27), 7023-7027. DOI: 10.1002/anie.201300322.

(23) Descalzo, A. B.; Ashokkumar, P.; Shen, Z.; Rurack, K. On the Aggregation Behaviour and Spectroscopic Properties of Alkylated and Annelated Boron-Dipyrromethene (BODIPY) Dyes in Aqueous Solution. *ChemPhotoChem* **2020**, *4* (2), 120-131. DOI: 10.1002/cptc.201900235.

(24) Bergström, F.; Mikhalyov, I.; Hägglöf, P.; Wortmann, R.; Ny, T.; Johansson, L. B. Å. Dimers of Dipyrrometheneboron Difluoride (BODIPY) with Light Spectroscopic Applications in Chemistry and Biology. *J. Am. Chem. Soc.* **2002**, *124* (2), 196-204. DOI: 10.1021/ja010983f.

(25) Pakhomov, A. A.; Kim, E. E.; Kononevich, Y. N.; Ionov, D. S.; Maksimova, M. A.; Khalchenia, V. B.; Maksimov, E. G.; Anisimov, A. A.; Shchegolikhina, O. I.; Martynov, V. I.; et al. Modulation of the photophysical properties of multi-BODIPY-siloxane conjugates by varying the number of fluorophores. *Dyes Pigm.* **2022**, *203*, 110371. DOI: 10.1016/j.dyepig.2022.110371.

(26) Austin, J.; Minelli, C.; Hamilton, D.; Wywijas, M.; Jones, H. J. Nanoparticle number concentration measurements by multi-angle dynamic light scattering. *J. Nanopart. Res.* **2020**, *22* (5), 108. DOI: 10.1007/s11051-020-04840-8.

(27) Sultanova, N.; Kasarova, S.; Nikolov, I. Dispersion Properties of Optical Polymers. *Acta Phys. Pol., A* **2009**, *116* (4), 585-587. DOI: 10.12693/APhysPolA.116.585.

(28) Armbruster, D. A.; Pry, T. Limit of blank, limit of detection and limit of quantitation. *Clin. Biochem. Rev.* **2008**, *29* (Suppl 1), S49.

(29) Maher, H. M.; Sultan, M. A.; Olah, I. V. Development of validated stability-indicating chromatographic method for the determination of fexofenadine hydrochloride and its related impurities in pharmaceutical tablets. *Chem. Centr. J.* **2011**, *5* (1), 76. DOI: 10.1186/1752-153X-5-76.

(30) Malothu, N.; Paladugu, T.; Katamaneni, P. Development and validation of rp-hplc method for determination of fexofenadine in pharmaceutical dosage form by using levocetirizine as an internal standard. *Int. J. Pharm. Biol. Sci.* **2018**, *8* (3), 619-625.

(31) Pinto, L. S. R.; Vale, G. T. d.; Moreira, F. d. L.; Marques, M. P.; Coelho, E. B.; Cavalli, R. C.; Lanchote, V. L. Direct chiral LC-MS/MS analysis of fexofenadine enantiomers in plasma and urine with application in a maternal-fetal pharmacokinetic study. *J. Chromatogr. B: Anal. Technol. Biomed. Life Sci.* **2020**, *1145*, 122094. DOI: 10.1016/j.jchromb.2020.122094.

(32) Sakur, A. A.; Nashed, D.; Noureldin, I. Green potentiometric determination of some of the third-generation antihistamines: Fexofenadine, Desloratadine, and Levocetirizine by using new carbon paste electrodes. *Talanta Open* **2022**, *5*, 100116. DOI: 10.1016/j.talo.2022.100116.

(33) Alrabiah, H.; Ali, E. A.; Alsalahi, R. A.; Attwa, M. W.; Mostafa, G. A. E. Fabrication and Applications of Potentiometric Membrane Sensors Based on γ-Cyclodextrin and Calixarene as Ionophores for the Determination of a Histamine H1-Receptor Antagonist: Fexofenadine. *Polymers* **2023**, *15* (13), 2808. DOI: 10.3390/polym15132808.

(34) Ashour, S.; Khateeb, M.; Mahrouseh, R. Extractive Spectrophotometric and Conductometric Methods for Determination of Fexofenadine Hydrochloride in Pharmaceutical Dosage Forms. *Pharm. Anal. Acta* **2013**, *S2*, 1-6. DOI: 10.4172/2153-2435.S2-003.

(35) Sharaf El-Din, M. K.; Ibrahim, F.; Eid, M. I.; Wahba, M. E. K. Validated Spectroflurimetric Determination of Some H1 Receptor Antagonist Drugs in Pharmaceutical Preparations Through Charge Transfer Complexation. *J. Fluoresc.* **2012**, *22* (1), 175-191. DOI: 10.1007/s10895-011-0944-x.

(36) Ramzy, S.; Abdelazim, A. H.; Hasan, M. A. Application of green first derivative synchronous spectrofluorometric method for quantitative analysis of fexofenadine hydrochloride and pseudoephedrine hydrochloride in pharmaceutical preparation and spiked human plasma. *BMC Chem.* **2022**, *16* (1), 62. DOI: 10.1186/s13065-022-00855-5.

(37) Hamad, A. A. Novel nano-leveled green switch-off fluorimetric technique for the determination of fexofenadine drug using Cilefa Pink B, a biological dye; raw material, dosage forms, and in vitro application; system kinetic study. *Talanta Open* **2022**, *6*, 100156. DOI: 10.1016/j.talo.2022.100156.

(38) Darwish, I. A.; Al-Outaibi, M.; Alzoman, N. Z. Tuning fluorescence of fexofenadine by switching OFF intramolecular photoinduced electron transfer: Application to development of one-step green and high throughput microwell spectrofluorimetric assay for analysis of tablets and plasma. *Luminescence* **2024**, *39* (7), e4818. DOI: 10.1002/bio.4818.

(39) Climent, E.; Biyikal, M.; Gawlitza, K.; Dropa, T.; Urban, M.; Costero, A. M.; Martínez-Máñez, R.; Rurack, K. A Rapid and Sensitive Strip-Based Quick Test for Nerve Agents Tabun, Sarin, and Soman Using BODIPY-Modified Silica Materials. *Chem. Eur. J.* **2016**, *22* (32), 11138-11142. DOI: 10.1002/chem.201601269.
